# Comparison Between Emerging and Conventional Methods for Edible Oils Bleaching

**DOI:** 10.1002/fsn3.71121

**Published:** 2025-10-29

**Authors:** Elahe Abedi, Hamid‐Reza Akhavan, Seyed Mohammad Bagher Hashemi, Najmeh Oliyaei, Mahmoud Sourghali, Ali Karimzadeh, Marzieh Rownaghi

**Affiliations:** ^1^ Department of Food Science and Technology, Faculty of Agriculture Fasa University Fasa Iran; ^2^ Department of Food Science and Technology, Faculty of Agriculture Shahid Bahonar University of Kerman Kerman Iran; ^3^ Department of Food Science and Technology, School of Agriculture Shiraz University Shiraz Iran; ^4^ Department of Remote Sensing and GIS, Faculty of Planning and Environmental Science Tabriz University Tabriz Iran; ^5^ Department of Mechanical Engineering, College of Engineering Fasa University Fasa Iran

**Keywords:** cost–benefit, friendly environment, novel bleaching method, reduced clay consumption

## Abstract

The activated bleaching clay is used at high temperatures and for extended periods to eliminate pigments and remove impurities from oils through physical and chemical interactions. However, the use of acid‐activated clay in industrial oil bleaching (IB) presents several drawbacks: prolonged filtration times due to the clay's fine particle size and compact structure; substantial oil loss and the generation of significant acid and acidic salts requiring specialized disposal; increased environmental concerns and landfill costs due to excessive clay use; degradation of triacylglycerols into free fatty acids (FFAs); rising oil acidity; formation of undesirable byproducts such as conjugated dienes and trienes; and generation of oxidation byproducts during bleaching due to high acid‐activated clay usage. Therefore, utilizing novel technologies to replace industrial approaches is of interest. Recently, ultrasound (US), microwave (MW), high‐voltage electric field (HVEF), and membrane (MB) assisted bleaching have attracted much attention. These innovative methods can enhance the adsorbents' sorption capacity, reduce the quantity of adsorbent required, and decrease the time and temperature needed, making them likely to be cost‐effective. In this review, the function of the industrial bleaching method for removing pigments, tocopherols, sterols, heavy metals, and primary and secondary oxidative products was investigated and compared with the emerging approaches. Adsorption isotherms favor the Freundlich and Langmuir models, reflecting heterogeneous multilayer adsorption. Kinetic studies often follow pseudo‐first‐order models for physisorption or pseudo‐second‐order for chemisorption, with intraparticle diffusion as a rate‐limiting step. Thermodynamic analyses indicate that these processes are endothermic and spontaneous, driven by entropy gains.

## Graphical abstract

The use of acid‐activated clay in industrial oil bleaching presents several drawbacks: prolonged filtration times, substantial oil loss, increased environmental concerns, landfill costs, degradation of triacylglycerols, increasing oil acidity, and formation of undesirable byproducts such as conjugated dienes and trienes. Utilizing novel technologies to replace industrial approaches is of interest. Recently, ultrasound, microwave, high‐voltage electric field, and membrane‐assisted bleaching have attracted much attention. These innovative methods could enhance the adsorbents' sorption capacity and reduce the adsorbent, time, and temperature.

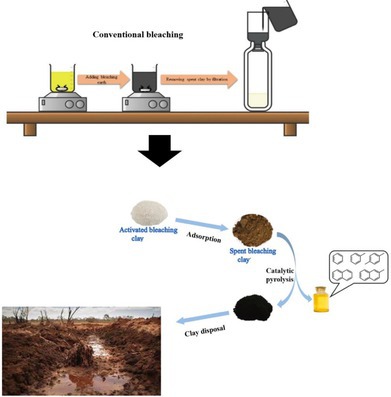

## 1 Introduction

The refining process is necessary to eliminate undesirable and toxic compounds present in crude oil. Many crude oils, referred to as “virgin oils,” exhibit characteristics such as an unpleasant appearance, off‐flavors, and susceptibility to oxidation, rendering them unsuitable for direct use in the production of consumer goods. Refining addresses these limitations, yielding products with improved sensory attributes and enhanced oxidative stability (Gharby 2022).

Bleaching is crucial in oil refining (following degumming, neutralization, and drying). It employs heat, chemical oxidation, and adsorption to remove undesirable pigments (carotenes and chlorophylls) and impurities (including metals, soaps, and oxidation products) (Łaska‐Zieja et al. 2020).

In the oil industry, bleaching clay is the predominant adsorbent material utilized in the decolorization of oils (Hussin, Aroua, & Daud, et al. 2011; More and Gogate 2018; Proctor and Brooks 2005). A comprehensive review by Hussin et al. (2011) and Abdelbasir et al. (2023) was compiled on the physical and chemical modification techniques of bleaching clay to investigate their effects on the structure, surface chemistry, and adsorption capacity. The adsorption process involves the attachment of contaminants to the surface of activated clay through two mechanisms: chemisorption and physisorption. Chemisorption occurs when the contaminant bonds with the activated clay surface through ionic or covalent bonding. This bonding results from the electron exchange between the sorbent surface and the contaminant. Chemisorption involves more significant changes in the electronic structure of both the sorbent and the contaminant, resulting in a stronger and more permanent attachment. On the other hand, physisorption is driven by van der Waals forces. In this process, the contaminant is attracted to the activated clay surface through weak intermolecular forces, namely London dispersion forces, dipole–dipole interactions, or hydrogen bonding. Physisorption does not involve substantial manipulation of the electronic structure of the sorbent or the contaminant and is generally weaker and reversible (Didi et al. 2009). The surface attraction forces, known as “van der Waals” forces, are responsible for the physical adsorption of carotenoid pigments in edible oils onto the surface of bleaching clays. Other constituents are chemically attached to the surface of the bleaching clay through covalent or ionic bonds. Certain impurities are removed via molecular entrapment within the porous structure of the clays. Certain minor constituents undergo chemical transformation during the bleaching process due to the catalytic activity of the clays. A well‐known example is the breakdown of hydroperoxides, resulting in the formation of unsaturated conjugated products (Abedi, Amiri, and Sahari 2020; Vaisali et al. 2014).

**FIGURE 1:**
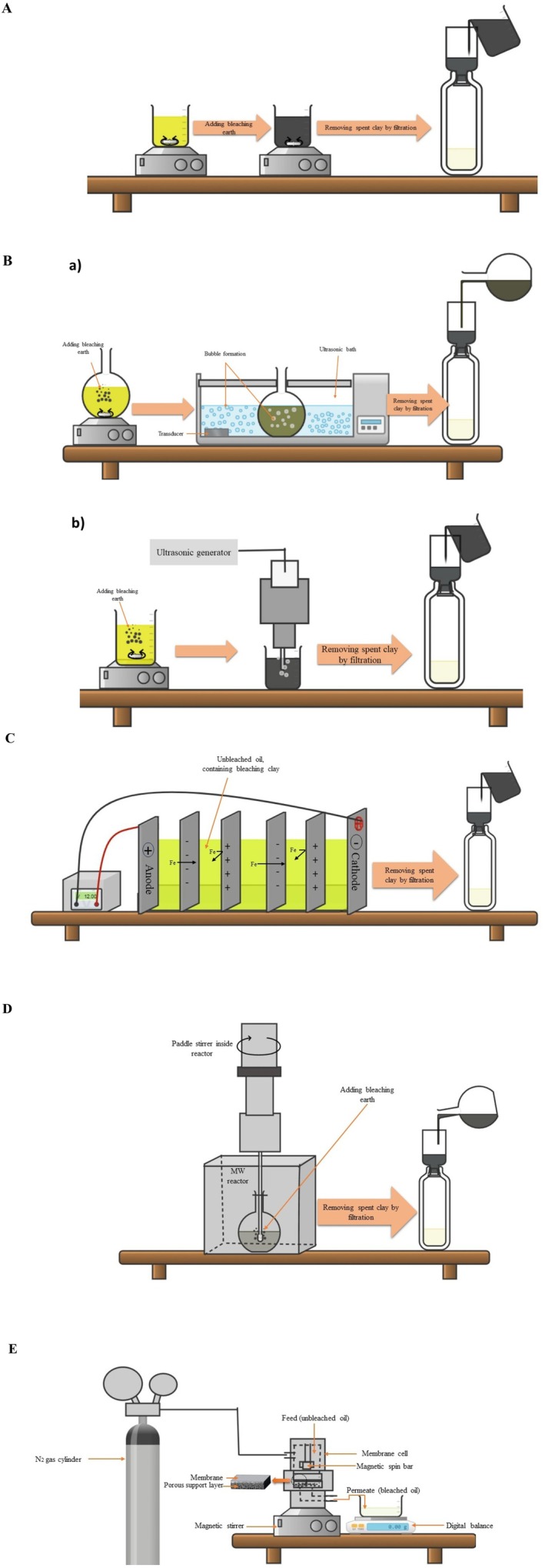
Conventional oil bleaching method (A), ultrasonic water bath (B‐a) and probe (B‐b)–assisted bleaching method, high voltage electrical field–assisted bleaching (C), microwave–assisted bleaching (D), and membrane–assisted edible oil bleaching method (E).

The widespread use of activated clays for bleaching edible oils in industrial bleaching presents several significant drawbacks. These include (A) prolonged filtration times due to the clays' fine particle size and compact structure; (B) substantial oil loss resulting from the clays' high oil retention capacity and also the production of significant amounts of acid and acidic salts requiring environmentally safe disposal methods (Hussin et al. 2011); (C) increased environmental concerns and landfill costs associated with their excessive use (Gunawan et al. 2010); (D) degradation of triacylglycerols into free fatty acids (FFAs), rising oil acidity (Abedi et al. 2015); (E) the formation of undesirable byproducts such as conjugated dienes and trienes, conversion of the *cis* configuration to *trans*, additional FFAs, and polymerization products of triacylglycerols (Abdelbasir et al. 2023; Dadfar et al. 2020); and (F) the generation of oxidation byproducts during bleaching due to the use of large quantities of acid‐activated clay (Seçilmiş et al. 2021a). Therefore, a comprehensive review article on the latest high‐tech approaches in oil bleaching techniques could be beneficial for reducing the use of bleaching clay and its impact on standard oil quality parameters (e.g., bio‐active retention, oil losses, oxidative indices). This review aims to compare industrial bleaching methods with ultrasonic‐assisted bleaching (UAB), microwave‐assisted bleaching (MWAB), high‐voltage electric field‐assisted bleaching (HVAB), and membrane‐assisted bleaching (MAB) to investigate the effects of each method on pigments, tocopherols, sterols, heavy metals, and primary and secondary oxidative products (Figure 1). Finally, the adsorption isotherms, kinetics, and thermodynamic studies were reviewed.

## 2 Industrial Oil Bleaching

Crude oils contain a variety of impurities, including plant pigments, phosphatides, oxidation products, and trace elements that affect the quality of edible oil. The bleaching step (decolorizing) is a complex physical and chemical process that is a crucial part of vegetable oil refining, which mainly eliminates pigments. Bleaching is carried out by mixing the clay adsorbents with the oil under specific conditions, and its efficacy is related to temperature, time, and the type and amount of bleaching clay (Figure 1A) (Seçilmiş et al. 2021b). Among various adsorbents, bleaching clay is widely used in industry and is the most common due to its low cost and high adsorption efficacy. Moreover, activated clays are another type of adsorbent that is activated through chemical reactions, such as acid treatments, which can increase clay porosity, binding sites, adsorption capacity, and surface area. Sometimes the mixture of bleaching clay and activated carbon is used to achieve better bleaching efficacy (Gharby 2022). The conventional (industrial) oil bleaching, employing 0.5%–2% clay at 90°C–120°C for > 30 min, relies on acid‐activated clay to maximize its surface area and impurity adsorption. While this improves color, increasing clay levels enhances this effect, increasing oil loss and generating environmental waste (Gupta 2017). Moreover, IB at higher clay levels can improve the color and appearance but cause higher oil loss and create environmental waste. Furthermore, using a high concentration of acid‐activated clay can promote oxidation reactions and the generation of oxidation products during IB (Seçilmiş et al. 2021a). Despite these limitations, IB remains widely adopted due to low equipment requirements, ease of control, and cost‐effectiveness at large scales.

### 2.1 The Effect of IB on Pigments and Colors

**FIGURE 2:**
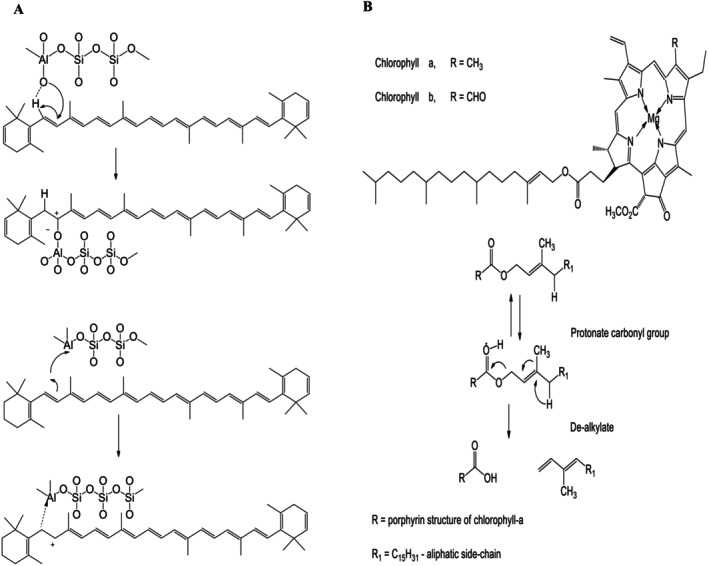
The mechanism of removal of carotenoid (A) and chlorophyll (B).

The predominant natural pigments in most vegetable oils are carotenoids and chlorophylls. Removing carotenoids, particularly β‐carotene, is crucial due to its impact on appearance. It has been reported that β‐carotene attaches to acid sites of the clay surface; therefore, acid activation is usually carried out for clays such as montmorillonite by physical and chemical interactions of its isoprenoid structure onto commercial bleaching clays (Ahmad et al. 2009; Chen and Sun 2023; Hambly et al. 2025). Figure 2A shows the mechanism of carotenoid adsorption on Bronsted and Lewis acid sites. As depicted, carotenoids are capable of binding effectively to the surface of the clay in the form of carbonium ions via coordinate bonds with Lewis sites or hydrogen bonds with Bronsted sites of the activated clay mineral (Hussin et al. 2011).

Figure 2B depicts the mechanism of chlorophyll removal. Chlorophylls comprise a cyclic tetrapyrrole structure chelated with a centrally located Mg^2+^ ion. Both chlorophyll a and chlorophyll b are the most prevalent types of chlorophyll found in vegetable oils. Chlorophyll b differs from chlorophyll a in that a formyl group replaces the C7‐methyl group. During oil processing, chlorophylls are converted to pheophytin by the loss of the central Mg^2+^ ion. It is worth noting that both chlorophylls and pheophytins are prooxidants that cause the production of singlet oxygen under light and promote oxidation reactions. Thus, the reduction of residual chlorophyll content can significantly improve oil stability (Chen and Sun 2023).

It has been reported that using 4% acid‐activated clay at 60°C and 80°C can reduce the carotenoid level of hemp (*Cannabis sativa L*.) oil by about 69% and 77%, respectively. At the same time, chlorophyll reduction was 72% and 77% under these conditions. It seems that a higher temperature was more effective in facilitating the interaction between bleaching clay and pigments (Chew and Ali 2021; Ferfuia et al. 2023; Jiang et al. 2020). Table 1 illustrates the reduction in chlorophyll and carotenoids in different oils bleached under various conditions.

**TABLE 1:** The effect of industrial bleaching (IB) on the bleached qualities of oils.

Type of oil	IB condition	Significant findings in ultrasonic treatment	Ref.
Palm oil	*Type of clay*: natural and acid‐activated bleaching earth *Amount of clay*: 0.5%–3.0% w/w *Temperature*: 105°C *Time*: 30 min *Pressure*: 50 mmHg	Acid‐activated bleaching earth was more effective than natural bleaching clay Decrease in carotenes from 258 mg/kg to 10 mg/kg by increasing acid‐activated earth from 0.5% to 3% Decrease in phosphorus (< 3 mg/kg) and iron (< 0.3 mg/kg) contents using 3% acid‐activated bleaching earth 1% acid‐activated and 2% natural earth was necessary for the reduction of PV value to zero	(Silva et al. 2014)
Hempseed and linseed oils	*Type of clay*: natural and acid‐activated *Amount of clay*: 2% and 4% *Temperature*: 60°C and 80°C	Decrease in carotenoid level (34%–42%) for natural bentonite Decrease in carotenoid level of hempseed oil after bleaching with 4% acid‐activated calcium bentonite by 69% and 77% at 60°C and 80°C, respectively Decrease in chlorophyll content of hempseed oil by 70% and 62% using 4% natural bentonite and acid‐activated earth at 80°C Bleaching with 4% natural bentonite at 80°C reduced 35% chlorophyll content of linseed oil Bleaching with 4% natural bentonite at 80°C reduced 62% chlorophyll content of hempseed oil Bleaching with 4% natural bentonite at 80°C reduced carotenoid content by 72% and 77% in linseed and hemp seed oils, respectively. Reduction in PV value by 14% and 10% in linseed and hempseed oils fatre bleaching with 4% natural bentonite at 80°C reduced PV value about 14% and 10%, respectively.	Ferfuia et al. (2023)
Safflower oil	*Temperature*: 90°C *Bleaching clay*: 0.50% Actisil +0.04% w/w Trisyl oil *Time*: 30 min	Increase in p‐anisidine value from 1.53 to 4.25 mmol/kg Decrease in tocopherol content from 249.8 ppm to 198.82 ppm	(Ortega‐García et al. 2006)
Olive and sunflower oils	*Conventional condition*: Bleaching clay: 1% w/w acid‐activated bleaching earth Temperature: 110°C Time: 30 min	Decrease in carotenoid content by 66.80% and 75.4% in olive and sunflower oils Decrease in chlorophyll content by 84.94% and 85.64% in olive and sunflower oils	(Abbasi et al. 2016)
Canola and corn oil	*Temperature*: 100°C–110°C *Bleaching earth*: 0.4%–0.8% *Time*: 30–45 min	Decrease in total sterol by 15.22% for canola oil and 42.57% for corn oil	(Vardin and Yorulmaz 2021)
Sunflower oil	*Bleaching clay*: 8 kg acid activated bleaching/1000 kg oil *Temperature*: 100°C under vacuum *Time*: 30 min	Decrease in chlorophyll (87.80%) Decrease in carotenoid (80.63%) Decrease in PV (23.3%) Decrease in □‐tocopherol (8%)	(Seçilmiş et al. 2021a)
Post‐fermentation corn oil	*Bleaching clay*: 2% of Calriant Tonsil 4120AFF, Clariant Supreme 112FF, Taiko Omega 1, Amcol Mineral Bent Actigel *Time*: 15 min *Temperature*: 60°C	Increase in acid value depending on bleaching clay Decrease in PV value Tonsil 4120AFF, Clariant Supreme 112FF caused the lowest PV value Decrease in phytosterols including campesterol (9.91%), stigmasterol (13.33%), □‐sitosterol (13.11%), and 5*σ*‐avenasterol (21.36%) after bleaching Decrease in carotenoid levels Decrease in red color	(Susik and Ptasznik 2023)
Soybean oil	*Clay*: Activated bentonite clay (0.5%, 1%, 1.5%, and 2% w/v) *Time*: 0–30 min *Temperature*: 35°C, 45°C, 55°C, and 65°C	Decrease in carotenoid content about 48% by increasing temperature from 35°C to 65°C Decrease in chlorophyll content about 38.9% by increasing temperature from 35°C to 65°C Decrease in iron and cupper levels about 3%–24% and 12.5%–24%, respectively by increasing temperature from 35°C to 65°C	(Abedi et al. 2021)
Distillers Corn Oil	*Bleaching clay*: 1%–3% *Time*: 5–25 min *Temperature*: 90°C–110°C	Optimum condition: 1.2% clay, 23.2 min, 99.1°C Decrease in □‐carotene content from 0.2% to 36.8% Decrease in red color from 27% to 88% Decrease in yellow color from 0% to 83% PV value was in range of 1.75 to 9.39 O_2_/kg	(Huda et al. 2024)

### 2.2 The Effect of IB on Oils' Bio‐Active Compounds (Sterols and Tocopherols)

Phytosterols are natural sterols that widely exist in plant oils and oilseeds and are primarily found as free sterols and as conjugates. Vegetable oils exhibit the highest concentration of total phytosterols, ranging from 150 to 1231 mg/100 g of oil (Bai et al. 2021). β‐sitosterol, campesterol, stigmasterol, and brassicasterol are the main phytosterols in edible oils (Yang et al. 2023). Phytosterols possess numerous biological properties, including anti‐diabetic, antioxidant, anti‐ulcerative, and anticancer effects. Phytosterols have a similar structure to cholesterol; therefore, they will compete in the intestine and reduce the absorption of cholesterol (Dikshit et al. 2020). Some studies confirmed phytosterols' role in reducing cholesterol absorption and improving the lipid profile; therefore, they have potential in alleviating cancer risk (Cabral and Klein 2017; El Omari et al. 2024).

The bleaching stage causes alterations of phytosterol oxidation products during the refining of crude oil. Competitive adsorption between phytosterols and pigments suggests that phytosterol loss is primarily due to the interactions between phytosterols and the adsorbent. Additionally, the pH value of adsorbent agents plays a role in forming phytosterol by‐products. Tocopherol degradation is affected by the bleaching clay's pH (Kreps et al. 2014). Moreover, the phytosterol esters content may decrease slightly after treatment with a bleaching agent. The acidity of the bleaching agent may contribute to the formation of steradienes and the hydrolysis of phytosterol esters (Verleyen et al. 2002). Furthermore, trace metal ions in bleaching clay can also contribute to phytosterol thermal oxidation, even at low concentrations (Bai et al. 2021).

Tocopherols, or vitamin E, are natural antioxidants commonly found in vegetable oils. While α‐, β‐, γ‐, and δ‐tocopherols all possess antioxidant properties, γ‐tocopherol demonstrates the most potent activity, followed by δ‐tocopherol. However, α‐tocopherol exhibits the highest activity as vitamin E (Gotor and Rhazi 2016). The bleaching process, however, reduces antioxidant compounds, notably tocopherols and phenolic compounds. It has been reported that bleaching with 2% natural bentonite decreased the tocopherols in linseed oil by approximately 26% (Table 1). Acid‐activated clay catalyzes tocopherol esterification but may also lead to the undesirable formation of 3‐monochloropropane‐1,2‐diol esters (Ferfuia et al. 2023). In this regard, Lucci et al. (2020) observed an 11% reduction of tocopherol content after bleaching of olive oil with a 3% bleaching clay mix containing 5% activated carbon at 90°C–97°C at 20 mbar. Susik and Ptasznik (2023) also investigated the type of bleaching clays (Calriant Tonsil 4120AFF, Clariant Supreme 112FF, Taiko Omega 1, Amcol Mineral Bent Actigel) on the reduction of β‐sitosterol content in post‐fermentation corn oil. The results showed that bentonite significantly reduced phytosterols, including campesterol (9.91%), stigmasterol (13.33%), β‐sitosterol (13.11%), and 5‐avenasterol (21.36%) after bleaching. The phytosterol content of post‐fermentation corn oil was reduced during acidic hydrolysis and dehydration of phytosterols.

### 2.3 The Effect of IB on Oxidative Indices

The oxidation of edible oils results in the generation of hydroperoxides, which represent primary oxidation products. During the bleaching process, the decomposition of hydroperoxides may generate a considerable quantity of secondary oxidation products, including aldehydes and ketones. Therefore, oil quality is estimated by determining the peroxide value (PV), thiobarbituric acid (TBA) value, and p‐anisidine value (p‐AV) (Bekdeşer et al. 2024). The formation of these particular compounds can be attributed to the activated bleaching clay properties. A negative correlation was identified between PV and the concentration of bleaching clay (Table 1). For instance, small amounts of bleaching clay can increase the secondary oxidation index. In contrast, a slight increase in clay concentration (greater than 1%) results in a reduction in PV to zero. Maximum p‐AV values coincide with the initial PV reaching zero, indicating the conversion of peroxides to secondary oxidation products (Silva et al. 2014). In this regard, Seçilmiş et al. (2021b) reported that the IB of sunflower oil, when treated with acid‐activated clay at 100°C for 30 min, reduced the toluene value of the bleached oil to 18.86 (equivalent to 6.63%) compared to before bleaching. Moreover, it has been reported that bleaching linseed and hempseed oils with 4% natural bentonite at 80°C resulted in a reduction of the PV by approximately 14% and 10%, respectively (Ferfuia et al. 2023).

### 2.4 The Effect of IB on Heavy Metals

Many edible oils contain heavy metal ions as impurities (Bekdeşer et al. 2024). The determination of heavy metals in oils presents challenging issues. This is due to the oil matrix's high viscosity and the challenges associated with oil oxidation, rancidity, and off‐flavor or flavor reversion (Szyczewski et al. 2016). During the bleaching step, adsorbents (or bleaching clay) attract undesirable compounds such as heavy metals and alter the composition of bleached oil (Table 1) (Huda et al. 2024). It has been reported that increasing the temperature from 35°C to 65°C during industrial bleaching of soybean oil can not only reduce carotenoid and chlorophyll levels but also decrease iron and copper content by 3%–24% and 12.5%–24%, respectively. Indeed, increasing the temperature by about 10°C can double the reaction rate and enhance the trace metal adsorption (Abedi et al. 2021). Silva et al. (2014) reported a reduction in phosphorus (to 2.7 ± 0.1 mg/kg) and iron (to 0.2 ± 0.1 mg/kg) contents after bleaching crude palm oil with 3% acid‐activated bleaching clay.

## 3 Novel Technological Oil Bleaching

### 3.1 Ultrasound‐Assisted Bleaching (UAB)

Ultrasound (US) is considered one of the most promising techniques for enhancing the bleaching efficiency of IB (Table 2). Diverse researches highlight the potential of US treatment as a sustainable and eco‐friendly method for oil bleaching, applicable both directly to oil samples and in conjunction with bleaching clay (Abedi et al. 2015; Chew and Ali 2021). The application of US technology has been investigated by several researchers in olive oil (Asgari et al. 2017, 2018; Jahouach‐Rabai, Trabelsi, et al. 2008a), soybean oil (Abedi et al. 2015; Abedi et al. 2017; Abedi et al. 2021, 2024; Abedi, Roohi, et al. 2020; Maleki et al. 2024; Roohi et al. 2019; Sayadi et al. 2024), sunflower oil (Maleki et al. 2024; Sayadi et al. 2024), hempseed oil (Aachary et al. 2016), and canola or rapeseed oils (Icyer and Durak 2018; Su et al. 2013).

**TABLE 2:** The effect of ultrasound‐assisted bleaching (USAB) and industrial oil bleaching (IB) on the bleached qualities of oils.

Type of oil	USAB condition	Major findings	IB	Ref.
Canola oil	*Temperature*: 45°C–75°C *Ultrasonic power*: 45–200 W *Ultrasonic mode*: Horn *Bleaching earth*: 0.1%–0.2% *Contact time*: 2–15 min	Reduction of oil temperature by 25% Reduction of contact time by 50% Reduction of red color by 55.8% Reduction of yellow color by 84.2% Reduction of PV value by 44.4% Increase in FFA value by 11.2% Increase in p‐Anisidine value 121.6% Reduction of conjugated dienes (K_232_) by 8.6% Increase in conjugated trienes (K_268_) by 75% Increase in totox by 10.12%	Reduction of red color by 58.82% Reduction of yellow color by 78.58% Reduction of PV value by 50.79% Increase in FFA value by 10.48% Increase in p‐Anisidine value 112.9% Reduction of conjugated dienes (K_232_) by 27.14% Increase in conjugated trienes (K_268_) by 225% Increase in totox by 1.5%	(Icyer and Durak 2018)
Canola oil	*Ultrasonic power*: 600 W *Frequency*: 40 kHz *Ultrasonic mode*: Horn *Bleaching earth*: 1%, 2%, and 3% *Temperature*: 60°C and 80°C *Contact time*: 60, 75, and 90 min	Reduction of chlorophyll *a* up to 96% Reduction of chlorophyll *b* up to 99% Reduction of β‐carotene up to 26%	Reduction of chlorophyll a up to 93% Reduction of chlorophyll b up to 80% Reduction of β‐carotene up to 29%	(De Jesús‐Hernández et al. 2023)
Olive oil	*Frequency*: 20 kHz *Ultrasonic Power*: 750 W *Bleaching earth*: 1% Tunisian acid‐activated clay *Amplitude*: 28% *Temp*: 60°C and 80°C *Time*: 13–45 min	Reduction of the volatile compounds and off‐flavor Reduction of sterols up to 14.44% Reduction of α‐tocopherols by 65.46% Increase in FFA by 0.16% Increase in PV by 16.4% Increase in phosphor by 300% Increase in iron by 54.5% Reduction of calcium by 64.2% Increase in magnesium by 150%	NR	
Olive oil	*Frequency*: 20 kHz *Ultrasonic Power*: 400 W *Ultrasonic mode*: Horn *Bleaching clay*: acid‐activated bentonite, 1% *Amplitude*: 30% *Temp*: 35°C–65°C *Time*: 0–30 min	Reduction of temperature by 35% Reduction of time by 57% Reduction of bleaching clay by 40% Reduction of tocopherols up to 931.8% Reduction of sterols up to 5.4% Reduction of iodine value by 0.19 Reduction of PV value by 44.48% Increase in TBA value by 20% Reduction of FFA by 25.49% Reduction of K_232_ by 5.65% Increase in K_270_ by 1383.3% Reduction of red color by 43.47% Reduction of yellow color by 57.42% Reduction of blue color Increase in L* by 8.6% Reduction of a* by 99.4% Reduction of b* by 68.8% Reduction of chlorophyll by 86.4% Reduction of carotenoids by 87.97%	Reduction of tocopherols up to 848.3% Reduction of sterols up to 5.6% Reduction of iodine value by 0.09% Reduction of PV value by 72.7% Reduction of TBA value by 20% Reduction of FFA by 32.54% Reduction of K_232_ by 27.7% Increase in K_270_ by 1850% Reduction of red color by 42.8% Reduction of yellow color by 60.3% Reduction of blue color Increase in L* by 12% Reduction of a* by 69.14% Reduction of b* by 80% Reduction of chlorophyll by 88.5% Reduction of carotenoids by 94.2%	(Asgari et al. 2017, 2018)
Olive oil	*Frequency*: 20 kHz *Ultrasonic Power*: 750 W *Ultrasonic mode*: Horn *Bleaching clay*: acid‐activated bentonite, 1% *Amplitude*: 28% *Temp*: 30°C–70°C *Time*: 13–45 min	Loss of α‐tocopherols up to 90.2% Total polyphenols loss up to 91.83% Reduction in phosphors by 93% Reduction in iron by 41.12% Reduction in calcium by 48.91% Increase in magnesium by 43.66%	Increasing bleaching index up to 80.9% Reduction of chlorophylls by 93.4% Loss of α‐tocopherols up to 27.2% Reduction of oxidative stability index up to 6% Reduction in phosphors by 62.7% Reduction in iron by 68.54% Reduction in calcium by 71.37% Reduction of magnesium by 54.92%	(Gharsalli et al. 2025)
Olive oil	*Frequency*: 20 kHz *Ultrasonic Power*: 750 W *Ultrasonic mode*: Horn *Bleaching earth*: bentonite, 1% *Amplitude*: 25%–75% *Temp*: 30°C *Time*: 30–60 min	Increasing bleaching index up to 86% Reduction of chlorophylls by 91.1% Loss of α‐tocopherols up to 90.2% Reduction of oxidative stability index up to 55% Reduction in phosphors by 93% Reduction in iron by 41.12% Reduction in calcium by 48.91% Increase in magnesium by 43.66%	Increasing bleaching index up to 80.9% Reduction of chlorophylls by 93.4% Loss of α‐tocopherols up to 27.2% Reduction of oxidative stability index up to 6% Reduction in phosphors by 62.7% Reduction in iron by 68.54% Reduction in calcium by 71.37% Reduction of magnesium by 54.92%	(Essid et al. 2016)
Olive and sunflower oils	*Frequency*: 20 kHz *Power*: 150 W *Ultrasonic mode*: bath *Adsorbent*: Activated bentonite *Adsorbent amount*: 0.1, 0.2, 0.5, 1, 2, and 5 g *Temperature*: 45°C and 60°C *Time*: 20 and 30 min	Reduction of carotenoids up to 79.7% (olive oil) Reduction of carotenoids up to 77.1% (sunflower oil) Increase in peroxide value in up to 33.3% (olive oil) Increase in peroxide value in up to 56.2% (sunflower oil) Reduction of acid value up to 59.1% (olive oil) Reduction of acid value up to 86.99% (sunflower oil)	Reduction of carotenoids up to 66.8% (olive oil) Reduction of carotenoids up to 75.4% (sunflower oil) Increase in peroxide value up to 120% (olive oil) Increase in peroxide value up to 34.37% (sunflower oil) Reduction of acid value up to 97.7% (in olive oil) Reduction of acid value up to 24.56% (sunflower oil)	(Abbasi et al. 2016)
Rapeseed oil	*Frequency*: 20 kHz *Power*: 750 W *Ultrasonic mode*: Horn *Adsorbent*: Activated alumina, zeolite, sepiolite, and bentonite *Adsorbent amount*: 0.1, 0.2, 0.5, 1, 2, and 5 g *Temperature*: 5°C, 25°C, 45°C, 65°C, and 86°C *Amplitude*: 20%, 40%, and 60% *Time*: 20 min	The higher the adsorption capacity of bentonite than activated alumina, zeolite powder, and sepiolite Pigment adsorption and degradation after ultrasonication Increasing the content of primary oxidation products The constant rate of secondary oxidation products Reduction of the A_446_ by 64.28%	NR	(Su et al. 2013)
Hempseed oil	*Bleaching clay*: 40 g/kg *Time*: 20 min *Power*: 20%, 40%, and 60%	Reduction of chlorophyll around 99.4% (industrial clay) > 97.8% (activated bentonite) > 82.7% (sepiolite) > 47.1% (non‐activated bentonite). Reduction of total phenolic content as 27.3% (industrial clay) > 33.4% (activated bentonite) > 34.7% (sepiolite) > 27.9% (non‐activated bentonite). Reduction of peroxide value to 88.5% industrial clay > 62.5% sepiolite > 25% non‐activated bentonite	Reduction of chlorophyll by 52.8% (sepiolite) > 52.1% (activated bentonite) > 15.3% (industrial clay) > 1.3% (non‐activated bentonite). Reduction of total phenolic content by 21.96% (sepiolite) > 21.1% (non‐activated bentonite) > 20.9% (activated bentonite) > 12.6% (industrial clay)	(Aachary et al. 2016)
Soybean oil, Sunflower oil, Canola oil, Olive oil and Palm oil	*Power*: 400–1000 W *Mode*: bath and horn *Amplitude*: 50%–100% *Temperature*: 35°C, 50°C, and 65°C *Time*: 10, 15, 20, 25, and 30 min *Frequency*: 25, 40, and 55 kHz *Bleaching clay*: bentonite; 0.5%–2%	Reduction of temperature by 35% Reduction of time by 10% Reduction of bleaching clay by 35% Reduction of carotenoids up to 83.6% (soybean oil) Reduction of carotenoids up to 63% (sunflower oil) Reduction of chlorophyll up to 95.24% (soybean oil) Reduction of chlorophyll up to 62% (sunflower oil) Reduction of red color up to 86.74% (soybean oil) Reduction of red color up to 100% (sunflower oil) Reduction of red color up to 100% (canola oil) Reduction of red color up to 100% (olive oil) Reduction of red color up to 69.58% (palm oil) Reduction of yellow color up to 83.71% (soybean oil) Reduction of yellow color up to 96.53% (sunflower oil) Reduction of yellow color up to 78.80% (canola oil) Reduction of yellow color up to 67.91% (olive oil) Reduction of yellow color up to 63.55% (palm oil) Reduction of peroxide value up to 74.66% (soybean oil) Reduction of peroxide value up to 56.2% (sunflower oil) Increase in TBA up to 66.7% (soybean oil) Reduction of TBA up to 87.5% (sunflower oil) Reduction of acid value up to 79.16% (soybean oil) Reduction of acid value up to 6.25% (sunflower oil) Reduction in phosphors by 85.3% (soybean oil) Reduction in phosphors by 97.6% (sunflower oil) Reduction in iron ND (soybean oil) Reduction in iron ND (sunflower oil) Loss of tocopherols by 43.52% (soybean oil) Loss of tocopherols by 32.53% (sunflower oil) Loss of sterols by 12.54% (soybean oil) Loss of sterols by 10.73% (sunflower oil)	Reduction of carotenoids up to 86.37% (soybean oil) Reduction of carotenoids up to 81.3% (sunflower oil) Reduction of chlorophyll up to 93.81% (soybean oil) Reduction of chlorophyll up to 49.15% (sunflower oil) Reduction of red color up to 67.81% (soybean oil) Reduction of red color up to 47.82% (sunflower oil) Reduction of yellow color up to 63% (soybean oil) Reduction of yellow color up to 66.93% (sunflower oil) Reduction of peroxide value up to 79.46% (soybean oil) Reduction of peroxide value up to 48.36% (sunflower oil) Reduction of TBA up to 16.7% (soybean oil) Reduction of TBA up to 92.5% (sunflower oil) Reduction of acid value up to 70.8% (soybean oil) Reduction of acid value up to 0% (sunflower oil) Reduction in phosphors by 87.5% (soybean oil) Reduction in phosphors by 97.4% (sunflower oil) Reduction in iron by 95.83% (soybean oil) Reduction in iron by ND (sunflower oil) Loss of tocopherols by 14.12% (sunflower oil) Loss of sterols by 2.6% (soybean oil) Loss of sterols by 4.25% (sunflower oil)	(E. Abedi et al. 2017; Abedi et al. 2015; Abedi, Roohi, et al. 2020; Abedi et al. 2021), (Sayadi et al. 2024), (Maleki et al. 2024)
Rice bran oil	*Power*: 50 W *Mode*: bath *Amplitude*: 50%–100% *Temperature*: 35°C, 50°C, and 65°C *Time*: 10 min *Frequency*: 25, 40, and 55 kHz *Bleaching clay*: Florisil, Magnesium trisilicate, Activated blending earth, Silica gel, Activated carbon, Calcium sulfate, and CK (20%)	Reduction of a* by 65.98% Reduction of carotenoids by 58.6% Reduction of acid value by 41% Reduction of peroxide value by 16% Increase in saponification value by 7.5% Increase in iodine value by 4.7% Reduction of oryzanol by 15.95% Reduction of squalene by 7.5% Reduction of tocopherol by 10.14% Reduction of polyphenol by 21.55% Reduction of campesterol by 0.3% Increase in β‐Sitosterol by 0.2% Reduction of stigmasterol by 0.4%	Reduction of a* by 63.92% Reduction of carotenoids by 38.95% Reduction of acid value by 30% Reduction of peroxide value by 43% Increase in saponification value by 8.4% Increase in iodine value by 6.9% Reduction of oryzanol by 9.9% Reduction of squalene by 3.6% Reduction of tocopherol by 5.4% Reduction of polyphenol by 14.53% Reduction of campesterol by 2.3% Increase in β‐Sitosterol by 0.8% Reduction of stigmasterol by 0.13%	(Zhang et al. 2024)

Abbreviations: ND, not detected; NR, not reported.

Using UAB offers several advantages, including enhanced bleaching efficiency, reduced consumption of bleaching clay, shorter processing times, and lower bleaching costs and losses.

Figure 1B shows the mechanism of the US bath and probe‐assisted bleaching methods. The enhanced adsorption effectiveness of ultrasonication is attributed to the mechanical effects of high‐intensity ultrasonic waves.

In a study by Abedi et al. (2024) and Essid et al. (2016), the treatment of bleaching clay with ultrasonication led to a significant increase in specific surface area and total pore volume, indicating an enhanced adsorption capacity. During UAB, cavitation near the particles creates surface cracks, erosion, and particle breakdown, resulting in a higher surface area and enhanced mass transfer (Abedi et al. 2024, 2020; Essid et al. 2016). This was observed across various power levels while bath ultrasonication was also tested. The efficiency of the ultrasonic treatment was strongly dependent on parameters such as amplitude, power, cycle time, temperature, and duration (Abedi, Amiri, and Sahari 2020; Chew and Ali 2021; Roohi et al. 2019; Su et al. 2013).

#### 3.1.1 Effects of Ultrasonication Parameters on Bleaching Efficiency

##### 3.1.1.1 Effect of Power

The enhanced bleaching efficiency observed with higher ultrasonic power intensifies results from cavitation‐induced phenomena. The intense implosion of cavitation bubbles creates increased fluid turbulence, microstreaming, and microjets, promoting dynamic adsorbent‐adsorbate interactions, increasing the number of adsorption sites, mass transfer, and generation of free radicals, thereby significantly improving the physical and chemical adsorption mechanism (Abedi et al. 2024, 2020; Asgari et al. 2017, 2018; Roohi et al. 2019). Moreover, the generated heat due to the boundary friction by ultrasonic waves increases the thermal energy and mass transfer (Hamidi et al. 2014). Therefore, a higher US amplitude results in the degradation of color pigments and improved bleaching efficiency (Abedi et al. 2015; Su et al. 2013; Vajnhandl and Le Marechal 2007). A numerical study revealed that a higher cavitation pressure led to higher collapse temperatures and more efficient bubble implosion (Roohi et al. 2019). The flow patterns generated by ultrasonic horns at 200 W/25 kHz and 400 W/40 kHz were analyzed. Both settings produced a complex flow: a descending plume, circulatory flow, and an upward wall plume. While higher power increased plume velocity, higher frequency led to faster energy dissipation. This flow significantly influenced the system's heat and mass transfer efficiency (Roohi et al. 2019).

##### 3.1.1.2 Effect of Frequency

Several factors contribute to the increased cavitation yield at lower US frequencies than higher frequencies: (1) High‐frequency sound waves experience more significant attenuation and scattering, reducing bleaching efficiency. (2) Lower frequencies allow larger bubbles (60–100 μm) to lead to faster cavitation formation and more vigorous collapse. (3) High frequencies restrict water vapor uptake during bubble expansion, reducing water vapor within collapsing bubbles. This decreases hydroxyl radical production, diminishing sonochemical and microstreaming effects (Abedi et al. 2017; Roohi et al. 2019).

##### 3.1.1.3 Effect of Temperature

Temperature can have both positive and negative effects on bleaching efficiency. The authors reported a twofold increase in reaction rate for every 10°C temperature increase, consistent with the van't Hoff rule. Generally, the lower temperature (< 65°C) for UAB can be selected for several reasons: (i) higher temperatures promote rapid bubble formation and growth, but the increased internal vapor pressure cushions the bubble collapse, reducing bleaching efficiency and potentially masking any sonochemical effects, (ii) high temperatures increase off‐flavor and rancid odor production during UAB, and (iii) lower temperatures also offer energy savings (Abedi et al. 2015). However, increasing temperature induces a lower viscosity and simplifies cavitation collapse.

##### 3.1.1.4 Effect of Oil Composition

Determining the viscosity of vegetable oil is essential for designing and optimizing flow and heat transfer unit operations during UAB. The oils' viscosities followed the order palm > olive > canola > sunflower > soybean. Viscosity is influenced by temperature and fatty acid composition (Fasina and Colley 2008; Santos et al. 2005). Viscosity rises with increasing molecular weight but reduces with increasing unsaturation. Because viscous liquids possess stronger cohesive forces, cavitation is more difficult to initiate in these liquids. Consequently, higher‐intensity sound waves or longer sonication times are needed to overcome the increased resistance to cavitation in viscous media (Abedi, Amiri, and Sahari 2020; De Jesús‐Hernández et al. 2023; Hamidi et al. 2014). Therefore, cavitation is simpler in low‐viscosity oils, such as soybean oil, than in high‐viscosity oils, like palm oil. Temperature has a significant impact on viscosity. This temperature effect is attributed to increased molecular motion, weakening intermolecular forces, and facilitating flow.

##### 3.1.1.5 Effect of Dissolved Gas in the Ultrasonicated Medium

The presence of dissolved gases facilitates the nucleation of cavitation. The choice of gas has a significant influence on the efficacy of cavitation. The intensity of cavitation depends on both the thermodynamic properties and the solubility of the gas employed. Gases exhibiting a high specific heat ratio are generally associated with a more pronounced cavitational effect, attributed to the rapid implosion dynamics of the generated bubbles (Thompson and Doraiswamy 1999). Monatomic gases (e.g., argon, helium) demonstrate superior cavitation performance compared to diatomic gases (e.g., nitrogen), a consequence of their higher specific heat ratios (Ar >He > air > N_2_) (Thompson and Doraiswamy 1999). Furthermore, an elevated gas solubility within the reaction medium promotes cavitation by increasing the number of available nucleation sites (Thompson and Doraiswamy 1999). The solubility hierarchy of gases in oil is as follows: (Ar >air > N_2_ > He). The solubility of noble gases, such as helium and argon, is enhanced by atomic mass, making argon considerably more soluble than helium in both organic and inorganic solvents (Kharaka and Specht 1988). In a study conducted by Abedi et al. (2017), a decrease in color and pigment intensity was observed in the following order: Ar >air > N_2_ > He. Argon has been shown to diminish color and pigment intensity compared to other gases. This gas exhibits more substantial cavitation due to its higher specific heat ratio and better solubility in the oil. The specific heat ratio of air is identical to that of nitrogen; however, air exhibits more excellent solubility in the oil medium. Additionally, oxygen can enhance the generation of hydroxyl radicals, which are crucial in the reaction mechanism. While nitrogen's solubility in the oil medium is more pronounced than that of helium, the higher specific heat ratio of helium contributes to a reduction in the color of bleached soybean oil. However, this pattern changes as temperature, frequency, and duration increase. The extent of color reduction for diatomic gases is more significant than for monatomic gases when temperature and time are elevated. This trend can be attributed to two factors: (i) gas solubility shows a decreasing pattern with rising temperature, and (ii) pressure in the oil medium increases with temperature according to the ideal gas law (PV = nRT). The increase in pressure is more significant for diatomic gases, such as nitrogen and air, than for monatomic gases, like argon and helium. Generating highly reactive radicals is favored under high‐temperature and high‐pressure conditions (Abedi et al. 2017; Kharaka and Specht 1988).

#### 3.1.2 The Effects of UAB on Pigments and Colors

Increasing the ultrasonic amplitude led to improved free radical formation, resulting in reduced color, carotenoid, and chlorophyll content. Cavitation‐induced pyrolysis within bubbles may also contribute to the destruction of pigments. Moreover, the formation of free radicals over ultrasonic processing leads to the degradation and discoloration of chlorophylls and β‐carotene (Tiwari et al. 2008). In Table 2, the percent reduction of pigments using UAB was documented for different oils. The sonication of a solution that contains suspended bleaching clay (0.5%–2%) releases energy owing to the collapse of cavitation bubbles, which are created by high‐velocity collisions between particles. Therefore, ultrasonic bleaching can continuously generate fresh clay surfaces capable of absorbing oil color and impurities (Abbasi et al. 2016; Abedi et al. 2015; Liang et al. 2017).

#### 3.1.3 The Effect of UAB on Oils՚ Bio‐Active Compounds (Sterols and Tocopherols)

The application of the ultrasonic horn and bath notably decreased the overall sterols and tocopherols content in sunflower oil (Sayadi et al. 2024), soybean oil (Abedi et al. 2015), and olive oil (Asgari et al. 2017, 2018; Essid et al. 2016; Gharsalli et al. 2025; Jahouach‐Rabai, M. Trabelsi, et al. 2008a) than industrial bleaching. Sterols are susceptible to degradation during both industrial and ultrasonic bleaching. The observed decrease in sterol concentration may be a consequence of several chemical reactions, including isomerization, adsorption, hydrolysis, dehydration, and esterification. These reactions contribute to a higher proportion of non‐polar components and terpenes in the bleached oil (Sayadi et al. 2024).

Sayadi et al. (2024) found contrasting stability trends during ultrasonic processing of sunflower oil. Δ7‐avenasterol exhibited the most outstanding stability in the industrial bleaching process of sunflower oil, whereas campesterol demonstrated the lowest stability. In contrast, sitostanol displayed the best stability in the UAB by bath. Δ5‐avenasterol demonstrated the greatest stability when processed using the ultrasonic horn. In comparison, campesterol exhibited the lowest stability regardless of whether the ultrasonic bath or horn method was employed (Sayadi et al. 2024). However, Abedi et al. (2015) reported that the overall sterol content decreased as the amplitude increased (65%–85%) in soybean oil. Among various forms, Δ7‐avenasterol and Δ5‐avenasterol displayed the most significant level of sensitivity. The most substantial decline in sterol content occurred at amplitudes of 85% (43.87%; Δ5‐avenasterol; and 35.26% in Δ7‐avenasterol) and 65% (26.2% in Δ7‐avenasterol and 32.4%; Δ5‐avenasterol). Observed that a minimal decrease in the overall sterol content occurs due to bleaching, particularly when the UAB of olive oil is used for 13 min. This decrease can be attributed to the dehydration of sterols into steradienes or their degradation into compounds that induce rancidity. 3,5‐stigmastadienes were detected in the highest concentration when the oil underwent the most severe bleaching conditions. Stigmastadienes, particularly 3,5‐stigmastadienes, are dehydration derivatives of β‐sitosterol and are classified within the broader family of sterenes. These compounds are generated during the bleaching and deodorizing stages of the process. Bleaching vegetable oils using acid‐activated clays typically leads to a substantial increase in steradiene content. However, the extent of this increase is influenced by the specific bleaching conditions, including the application of ultrasound. The use of ultrasound is particularly beneficial because it prevents the dehydration of β‐sitosterol, the predominant sterol in the sterolic fraction, which is a common side reaction during bleaching. Bleaching clays exhibited significant catalytic activity in the formation of stigmastadiene. However, employing an ultrasonic bleaching process significantly reduced β‐sitosterol dehydration, although the degree of reduction decreased as bleaching time increased.

Numerous researchers' results indicated that both IB and UAB lowered the amount of tocopherol content in sunflower, soybean, and olive oils (Abedi et al. 2015; Asgari et al. 2017, 2018; Sayadi et al. 2024). IB reduced sunflower oil tocopherol content by 16.80%. UAB, however, showed significantly greater reductions: 23% (200 W horn), 21.3% (400 W bath), 37.8% (400 W horn), and 36.6% (800 W bath). This enhanced reduction is attributed to ultrasonic wave‐induced oxidation and free radical generation, which decompose tocopherols and impair their antioxidant properties (Sayadi et al. 2024). The lower reduction in total tocopherols observed with IB, followed by ultrasonic bath and ultrasonic horn bleaching at the same theoretical power, suggests that the horn method generates a higher concentration of free radicals. These free radicals are likely responsible for the increased degradation of tocopherol. Jahouach‐Rabai et al. (2008a) research showed a decline in the content of ‐tocopherol following the UAB. The significant reduction in tocopherol content can be attributed to their decomposition or oxidation, and conversion into various products, such as quinones, dimers, trimers, and epoxides, when tocopherols are subjected to severe bleaching conditions. Abedi et al. (2015) mentioned that after 20 min, the value of ‐tocopherol in the bleached soybean oil decreased in both the control and UAB processes. However, γ‐tocopherol and δ‐tocopherol increased by 1 and 1.5 times, respectively, exclusively during the UAB. The degree of hydroxylation can affect the antioxidant activity in both food and biological systems. Free radicals can potentially harm antioxidants such as phenols, reducing the bioactivity of various food components. However, an increase in hydroxylation may enhance the antioxidant activity of other compounds, such as flavonoids (Soria and Villamiel 2010).

After UAB, both total and individual tocopherols were markedly reduced. The reduction in α‐tocopherol under ultrasonic bleaching has been attributed to ultrasonic‐induced decomposition and oxidation (Asgari et al. 2017). Notably, differences among bleached samples were not significant for most tocopherols, except β‐tocopherol. According to the findings by Asgari et al. (2017), lower temperature, a 50% reduction in activated clay, and low‐frequency ultrasound—likely diminish radical formation and mitigate harmful ultrasonic effects, enabling the retention of substantial tocopherol quantities. Therefore, from a nutritional viewpoint, an ultrasonic bath could constitute a safer means of employing ultrasonic energy. The activation conditions of bleaching clays significantly impacted the retention of tocopherol in pomace olive oil. Using a very low acid concentration (0.5 M) during activation of clay with (50% ultrasound amplitude, 45 min) minimized tocopherol loss (22%). Conversely, higher acid concentrations (1.5 M and 2.5 M) during activation, coupled with lower ultrasound amplitude (28%), resulted in considerably higher tocopherol losses (34% and 62%, respectively) (Essid et al. 2016; Gharsalli et al. 2025). Moreover, Zhang et al. (2024) stated that the low micronutrient content (oryzanol, squalene, tocopherols, and polyphenols) of rice bran oil bleached with ultrasound may result from the action of ultrasound.

#### 3.1.4 The Effect of UAB on Oxidative Indices

Vegetable oil contains carotenes and tocopherols, which employ various quenching mechanisms to inhibit oxidation. During the UAB, an increase in PVs and TBAs was demonstrated in conjunction with a decrease in antioxidant concentration. For instance, Abedi et al. (2015); Gharsalli et al. (2025) and Sayadi et al. (2024) indicated that a decrease in tocopherol levels directly affects the oils' resistance to oxidation because a remarkable drop in the oxidation resistance of bleached oils is directly associated with decreased tocopherol content. Moreover, the medium's oxidative degradation by free radicals is the leading degradation mechanism. Furthermore, this rise in the amount of PV may result from the production of oxidation products occurring at a faster rate than the adsorption capacity of the bleaching clay after a 30‐min bleaching process (Abedi et al. 2015). Secondary oxidation byproducts, including aldehydes and ketones, possess volatility and are found to experience thermal or ultrasound decomposition during UAB. Su et al. (2013) demonstrated that PV was enhanced when a US 60% amplitude or heating at 200°C was used for bleaching without an adsorbent. Additionally, applying ultrasound at 40% amplitude in the presence of an adsorbent resulted in an increase in oxidative compounds. Nonetheless, secondary oxidation products remained unchanged across all experimental conditions.

#### 3.1.5 The Effect of UAB on Heavy Metals

The involvement of Fe and Cu in the oxidation process of vegetable oils is highly significant. The findings obtained by Abedi et al. (2015, 2021) showed that the utilization of US in the bleaching process resulted in a notable decrease in the levels of Fe and Cu compared to the IB method. This decrease might be attributed to the adsorption of these elements on the bleaching clay. Applying ultrasonic waves induces cavitation on the solid surface, which leads to the abrasion of the clay, providing a new surface for element absorption and facilitating the uptake of these elements. A comparable mechanism is noted in the absorption of phosphorus and soap onto the bleaching clay (Abedi et al. 2015, 2021).

#### 3.1.6 Effect of UAB on Clay Consumption

Utilizing UAB in oil bleaching can be an alternative method to IB because this technique can attenuate the bleaching conditions by reducing temperature and clay content. For instance, it was reported that the optimum condition for removing 98% of chlorophyll compounds was achieved using UAB with 2% clay at 60°C for 60–90 min. This method was more effective than the IB method, which used 3% bleaching clay at 100°C for 180 min (De Jesús‐Hernández et al. 2023). In a study by Abedi et al. (2015), UAB could reduce clay (35%), temperature (35%), and time (10%). Additionally, a reduction of temperature by 35%, time by 57%, and bleaching clay by 40% was achieved by Asgari et al. (2017, 2018).

**FIGURE 3:**
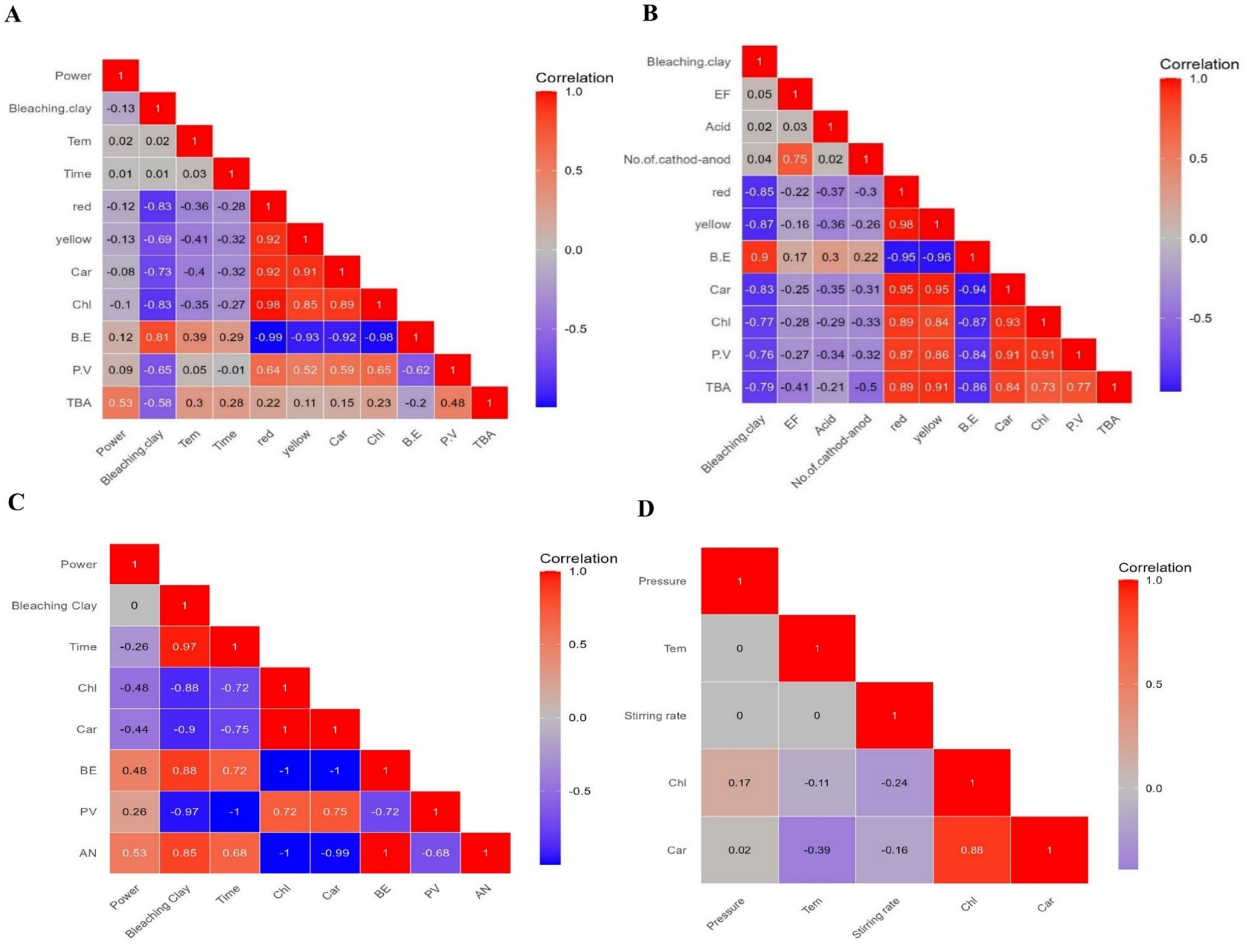
Heat maps of UAB (A), HVAB (B), MWAB (C), and MAB (D). AN, anisidine value; BE, bleaching efficiency; Car, carotenoid; Chl, chlorophyll; PV, peroxide value; TBA, thiobarbituric acid; Tem, temperature.

The provided heatmap (Figure 3) displays the correlation coefficients between various variables, revealing significant interrelations within the dataset. The color‐coded representation ranges from deep red (strong positive correlation) to blue (strong negative correlation). A noteworthy observation is the strong positive correlation between yellow (0.91 and 0.85) and red (0.92 and 0.98) with carotenoid and chlorophyll, respectively. Conversely, red and yellow depict a near‐perfect negative correlation with B.E (−0.99 and −0.93). In addition to these patterns, the effect of bleaching clay demonstrates a stronger positive correlation (0.81) with B.E. than ultrasonic power (0.12), emphasizing that the importance of bleaching clay is not inevitable for reducing pigments and colors.

### 3.2 High Voltage Electrical Field‐Assisted Bleaching (HVAB)

**FIGURE 4:**
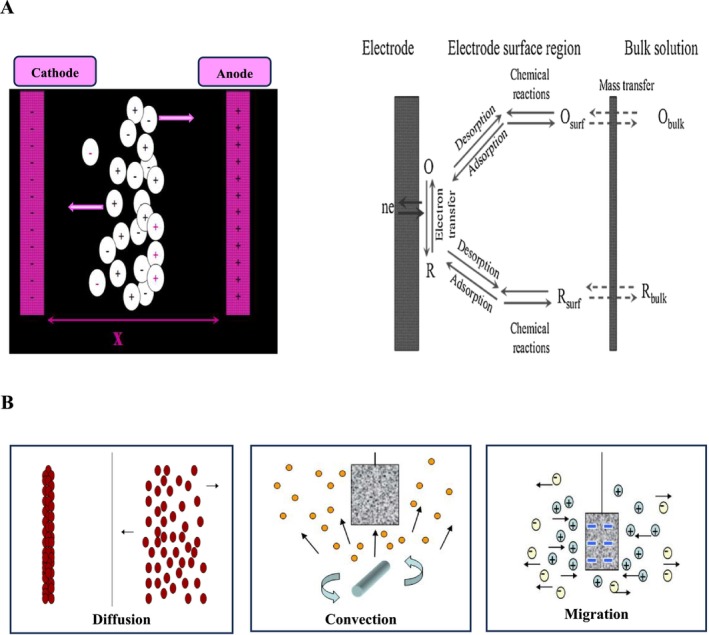
The mechanism of pigment removal by HVEF (A) mode of action of pigments adsorption on the electrode surfaces (B) mass transport mechanisms by diffusion, migration, and convection govern the separation of oil pigments.

HVEF is widely recognized as a primary non‐thermal processing technology. Figure 1C depicts the mechanism of HVAB in the bleaching of edible oils. The HVEF is recognized for its ability to introduce energy through a low‐current/high‐voltage electrical discharge between two electrodes. Electrosorption is typically the adsorption induced by an electric potential on charged electrode surfaces. The fundamental principle of electrosorption involves the movement of charged ions within an electrolyte solution toward electrodes of opposite charge, facilitated by the application of an electric field (Figure 4A, Table 3). Upon applying this field, high‐conductivity electrodes with a substantial surface area exhibit the formation of strong electrical double layers (Abedi et al. 2016, 2021; Mousavifard et al. 2024; Mwaurah et al. 2019). Three fundamental mass transport mechanisms govern the separation of oil pigments (Figure 4B):

**TABLE 3:** The effect of high voltage electric field‐assisted bleaching (HVEFAB) and industrial oil bleaching (IB) on the bleached qualities of oils.

Type of oil	HVEFAB condition	Major findings	IB	Ref.
Soybean oil and Sunflower oil	*Clay percentage*: (0.5%–2%) *Voltage*: (0 and 24 kV) *Distance*: (8‐12 mm) *Numbers of cathode–anode*: (4–8) *Temperature* (35°C–65°C), *Time* (0–30 min)	Reduction of a* by 61.73% (soybean) Reduction of a* by 139.58% (sunflower) Reduction of b* by 48.19% (soybean) Reduction of b* by 134% (sunflower) Reduction of red by 71.26% (soybean) Reduction of red by 143.47% (sunflower) Reduction of yellow by 63% (soybean) Reduction of yellow by 132.85% (sunflower) Reduction of carotenoids by 85.61% (soybean) Reduction of carotenoids by 118.7% (sunflower) Reduction of chlorophyll by 94.86% (soybean) Reduction of chlorophyll by 137.09% (sunflower) Reduction of peroxide value by 74.66% (soybean) Reduction of peroxide value by 136.8% (sunflower) Reduction of TBA value by 61.53% (soybean) Reduction of TBA value by 175% (sunflower) Reduction of FFA by 50% (soybean) Reduction of FFA by 89.4% (sunflower) Reduction of sterols by 8.8% (soybean) Reduction of sterols by 4.2% (sunflower) Reduction of tocopherols by 13.58% (soybean) Reduction of tocopherols by 171.4% (sunflower)	Reduction of a* by 58.11% (soybean) Reduction of a* by 48.26% (sunflower) Reduction of b* by 48.44% (soybean) Reduction of b* by 65.61% (sunflower) Reduction of red by 67.81% (soybean) Reduction of red by 47.82% (sunflower) Reduction of yellow by 60.71% (soybean) Reduction of yellow by 81.30% (sunflower) Reduction of carotenoids by 86.37% (soybean) Reduction of carotenoids by 81.30% (sunflower) Reduction of chlorophyll by 93.81% (soybean) Reduction of chlorophyll by 49.15% (sunflower) Reduction of peroxide value by 79.46% (soybean) Reduction of peroxide value by 67.56% (sunflower) Reduction of TBA value by 80.56% (soybean) Reduction of TBA value by 25% (sunflower) Reduction of FFA by 50% (soybean) Reduction of FFA by 175% (sunflower) Reduction of sterols by 2.6% (soybean) Reduction of sterols by 186.5% (sunflower) Reduction of tocopherols by 5.67% (soybean) Reduction of tocopherols by 14.12% (sunflower)	Abedi et al. (2019) Abedi, Roohi, et al. (2020) Abedi et al. (2016)

#### 3.2.1 Diffusion

Molecular diffusion is driven by concentration gradients. The rate is determined by the concentration difference between two points and the diffusion coefficient, a species‐specific value dependent on temperature (Equation 1). This process, described by Fick's first law, involves the movement of particles across a cross‐sectional area in response to the concentration gradient across that area (Bard et al. 2022).

(1) $$D={\mathrm{\delta C}}_{\left(x,t\right)}/\mathrm{\delta x}$$

δC_(x,t)_/δx, Concentration gradient (x and t are distance and time).

#### 3.2.2 Migration

The contribution of ionic migration to overall mass flux is proportional to the ion's charge, concentration, and diffusion coefficient, as well as the applied electric field gradient (Equation 2). Molecular weight also influences this contribution. Separately, altering the potential applied to a solid electrode in an ionic solution directly affects charge migration. Charged species move rapidly toward the electrodes, modulated by solution pH and ionic strength (Bard et al. 2022).

(2) $$\left[\left(\mathrm{zF}/\mathrm{RT}\right){\mathrm{DC}}_{\left(x,t\right)}\right]{\delta \phi }_{\left(x,t\right)}/\mathrm{\delta x}$$

z: Charge number of the ion (valence). This is the number of elementary charges carried by the ion. D: Diffusion coefficient. This describes how quickly a particle diffuses through a medium. It's dependent on the particle itself, the medium, and the temperature. Units are typically cm^2^/s or m^2^/s. F: Faraday constant. This is the charge carried by one mole of electrons. R: Ideal gas constant. This constant appears in many equations involving temperature and energy. T: Temperature in Kelvin. Absolute temperature is crucial in many physical and chemical processes. δϕ: Change in electric potential (or potential difference). This represents the difference in electrical potential between two points, often expressed in Volts. δx: Change in distance (or spatial difference).

#### 3.2.3 Convection

Stirring or other methods inducing bulk solution movement (convection) provide a controllable transport mechanism (Equation 3). The solution's hydrodynamic velocity (ν_x_) determines the one‐dimensional convective flux.

(3) $$C_{\left(x,t\right)}v_{x\;\left(x,t\right)}$$

C, Concentration (mol/cm^3^); V, Velocity (cm/s).

The Nernst‐Planck equation (Equation 4) was used to calculate the one‐dimensional mass flux to the electrode surface (Bard et al. 2022).

(4) $$J_{\left(x,t\right)}=-D\frac{{\mathrm{\delta C}}_{\left(x,t\right)}}{\mathrm{\delta X}}-\frac{\text{zFDC}}{\mathrm{RT}}\times \frac{{\delta \phi }_{\left(x,t\right)}}{\mathrm{\delta X}}+C_{\left(x,t\right)}V_{\left(x,t\right)}$$

δC _(x,t)_/δx, Concentration gradient (x and t are distance and time); δϕ _(x,t)_/δx, Potential gradient; J (x,t), Flux at × distance and time t; C, Concentration (mol/cm^3^); D, Diffusion coefficient (cm^2^/s); Z, Charge of species; V, Velocity (cm/s).

The mass flux comprises contributions from diffusion, migration, and convection, as represented by the three terms. These mechanisms facilitate the migration of polar and ionic compounds, such as pigments, FFAs, soaps, phosphates, trace elements, peroxides, and hydroperoxides, to the electrode surfaces under HVEF conditions.

During the HVEFAB technique, several key parameters, including the number of cathodes and anodes, voltage, and the use of an inert electrolyte, influence the bleaching yield. For instance, increasing the number of electrodes increases the surface area, resulting in higher fluid transfer across both the cathode and anode. Therefore, the use of numerous electrodes results in better discoloration and higher bleaching efficiency. Moreover, higher voltage and a few concentrations of inert electrolytes can boost pigment adsorption and bleaching efficiency. Inert electrolytes, such as HCl, are broken down into H^+^ and Cl^−^. These ions promote pigment movement across the electrodes because ions increase the charge density of pigments (Abedi et al. 2016). Abedi, Roohi, et al. (2020) reported that HVAB with 20 kV was more effective than 10 kV. Similarly, Tavakoli et al. (2019) also noted that the discoloration of pigments increased when the voltage was enhanced from 5 to 15 kV. Graphite's suitability as an adsorbent for the purification of vegetable oils stems from its favorable combination of properties: high electrical conductivity and thermal stability, substantial surface area (1–2000 m^2^/g), and low cost. Moreover, the treatment of activated carbon with KOH increases the percentage of carboxyl and carbonyl functional groups, leading to enhanced interaction and pigment adsorption. The temperature dependence of the bleaching process exhibits a near‐doubling of the reaction rate for each 10°C increment. An Arrhenius‐type relationship is consistently observed for the viscosity reduction (approximately 30% per 10°C increase), attributable to the weakening of intermolecular forces and increased molecular kinetic energy. This decrease in viscosity facilitates enhanced ion transport to the electrode surfaces (Abedi et al. 2016, 2020; Mousavifard et al. 2024).

#### 3.2.4 The Effect of HVAB on Pigments and Colors

HVAB has been used successfully for edible oil bleaching (Table 3). HVAB is a more suitable technique for reducing chlorophyll and carotenoids. Soybean and sunflower oils both contain carotenoids, primarily β‐carotene and α‐carotene. Soybean oil's carotenoid content is approximately 98% β‐ and α‐carotene, with the remaining 2% composed of minor carotenoids such as cryptoxanthin, zeaxanthin, and lutein. Sunflower oil is composed similarly of mostly β‐ and α‐carotene (95%), with the remaining 5% comprising other carotenoids, including canthaxanthin, lutein, and zeaxanthin. In contrast, chlorophyll contains a Mg^2+^ within a porphyrin ring and has a carboxyl side chain. Carotenoids and xanthophylls, characterized by cyclic structures that may or may not contain hydroxyl groups and possess multiple double bonds, exhibit migration toward the cathode and anode under HVEF conditions. The Nernst‐Planck equation indicates that pigment movement to the electrode surfaces is accelerated by increased voltage (20–24 kV) (Mousavifard et al. 2024). Additionally, using activated electrode surfaces at high voltage during bleaching can reduce the amount of adsorbent required, due to their ability to enhance the adsorption capacity of the clay. Its ability is attributed to the electric discharge, which can cause mechanical damage to clays and fragment particles. Thus, the generated smaller particles can create a high surface area and higher pigment adsorption (Abedi et al. 2016, 2020; Mousavifard et al. 2024). Therefore, the higher voltage during HVAB can extend mass transport, which causes excessive pigment elimination (Bard et al. 2022).

#### 3.2.5 The Effect of HVEF on Oils՚ Bio‐Active Compounds (Sterols and Tocopherols)

There is a rare investigation about the effect of HVAB on the sterol content of edible oils. The findings showed that sterol and tocopherol levels decreased to around 8.88% and 13.58% (for soybean) and 13.64% and 28.59% (for sunflower oil) after HVAB at 24 kV compared to IB, which resulted in reduced sterol and tocopherol levels (2.62% and 5.50% for soybean) and (4.24% and 14.12% for sunflower oil), respectively (Abedi et al. 2016, 2020; Mousavifard et al. 2024). Sterols and tocopherols are cyclic structures that may have hydroxyl groups and contain numerous double bonds. Under HVEF conditions, these sterols and tocopherols migrate toward the cathode and anode surfaces or decompose under a high‐voltage electric field.

#### 3.2.6 The Effect of HVEF on Oxidative Indices

Recently, it has been claimed that the HVAB can effectively adsorb polar compounds, including oxidative compounds, on the electrode surface. This feature is attributed to the chemical properties of peroxide compounds, which have a carboxyl or hydroxyl group in their molecules. The presence of two oxygen atoms bonded together (‐*O‐O*‐) in their structure facilitates transfer on the electrode surfaces during HVAB as polar groups (Mousavifard et al. 2024). The efficacy of HVAB at higher electrical voltages is more remarkable. In this regard, Tavakoli et al. (2019) reported that the PV of sunflower oil was reduced by approximately 48% when the electrical voltage was increased from 5 to 15 kV. Meanwhile, the comparison between the bleaching of soybean oil at HVEF‐10 kV and 20 kV showed that the PV values were 77.6% and 79.4%, and the TBA values were 50% and 33.3%, respectively. These values were more than those obtained using the IB method, with a PV value of 74.66% and a TBA value of 16.6% (Abedi, Roohi, et al. 2020). Similarly, Abedi et al. (2016) investigated the HVEF (0–24 kV) on the oxidative indices of soybean and sunflower oils. The authors observed that a 24 kV voltage with 1% clay at 65°C for 20 min was the optimum condition for soybean oil, reducing PV by approximately 81.06% and TBA by approximately 49.20%. However, the optimal condition for sunflower oil was 22 kV with 0.5% clay at 65°C for 20 min, resulting in a PV of approximately 81.19% and a TBA of roughly 54.54%.

#### 3.2.7 The Effect of HVEF on Heavy Metals

The mechanism for removing metals is related to the medium's ionic strength due to the presence of heavy metals such as Fe^2+^ and Cu^2+^, and convection boosts the metals' movements across the solution (Abedi et al. 2016, 2020; Mousavifard et al. 2024).

#### 3.2.8 Effect of HVEF on Clay Consumption

As previously mentioned, researchers have established that HVAB reduced clay content during the bleaching process (Abedi et al. 2016, 2020; Mousavifard et al. 2024). It has been reported that following HVAB, clay amount, temperature, and time declined by about 35%–45%, 50%, and 55%, respectively, which is due to the increased mass and heat transfer during the bleaching (Mousavifard et al. 2024). The presented heatmap illustrates a strong positive correlation (0.9) between bleaching clay and B.E, while a minor positive relationship exists between BE (0.17) and power. Similarly, red (−0.85) and yellow (−0.87) variables exhibit a negative correlation with (−0.22) and (−0.16) with bleaching clay and electrical power, implying a close relationship between color removal and bleaching clay (Figure 3).

### 3.3 Microwave‐Assisted Bleaching (MWAB)

The effectiveness of MWAB stems from rapidly heating materials with low specific heat, such as vegetable oils. It can provide some advantages in edible oil processing, reducing processing time and improving internal heat penetration (Gjorgjevich et al. 2012). Moreover, microwave treatment enhances the sorption capacity of clays, increasing the rate constant for sorption reactions by a factor of 107.6 and lowering the free energy. Furthermore, it does not alter the layered structure of bentonite clays (Seçilmiş et al. 2021a, 2021b).

#### 3.3.1 The Effect of MWAB on Pigments and Colors

Seçilmiş et al. (2021a) stated that in MW techniques, the reduction of chlorophyll was 85.37% for MWAB and 87.80% for IB of sunflower oil, respectively (Table 4). On the other hand, the carotenoid content experienced a decrease of 80.63% in the IB of sunflower oil and 74.87% in the MWAB of sunflower oil. The lower a* value and chlorophyll content of MWAB in sunflower oil compared to the IB method indicated that the MW process effectively diminished redness, implying a higher sorption capacity of clay after microwave heating. In IB and MWAB, the oil's yellowness value (b*) experienced a decline, while the yellowness is slightly higher in MWAB, which corresponds to a higher carotenoid content (Seçilmiş et al. 2021a, 2021b).

**TABLE 4:** The effect of microwave‐assisted bleaching (MWAB) and industrial oil bleaching (IB) on the bleached qualities of different types of oil.

Type of oil	MWAB condition	Major findings	IB	Ref.
Sunflower oil	*Power*: 80 W *Amount of clay*: 0.4% *Bleaching time*: 8 min *Temperature*: < 100°C	Reduction of a* by 557.10% Reduction of b* by 15.11% Reduction of carotenoids by 74.87% Reduction of chlorophyll by 85.36% Reduction of peroxide value by 6.26% Increase in P‐anisidine value by 857.5% Increase in Totox value by 10.84% Increase in FFA by 3.70% Increase in sterols by 0.43% Reduction of tocopherols by 7.79%	Reduction of a* by 450% Reduction of b* by 16.53% Reduction of carotenoids by 80.63% Reduction of chlorophyll by 87.80% Reduction of peroxide value by 23.33% Increase in P‐anisidine value by 820% Reduction of Totox value by 6.63% Reduction of FFA by 25.92% Reduction of sterols by 1.14% Reduction of tocopherols by 8%	(Seçilmiş et al. 2021a, 2021b).

#### 3.3.2 The Effect of MWAB on Sterols and Tocopherols

According to a study conducted by Seçilmiş et al. (2021a), no statistically significant differences were observed in sterol composition between unbleached, MWAB, and IB. However, the loss of tocopherol in MWAB for sunflower oil was statistically equivalent to that in the IB method. Javidipour et al. (2017) revealed that using MW treatment (600 W) for 9 min reduced the tocopherol content of sunflower oil by 16.85%. These results suggested that higher MW energy effectively declined tocopherol levels, while lower MW energy with a short time can preserve tocopherol content (Seçilmiş et al. 2021a, 2021b).

#### 3.3.3 The Effect of MWAB on Oxidative Indices

Seçilmiş et al. (2021a) revealed that although PV exhibited a reduction pattern in MWAB and IB methods for sunflower oil, no significant changes in the PV were observed in the MWAB process compared to unbleached oil. This can be attributed to using 50% less clay than the IB method and the shorter bleaching duration of MWAB. The absence of a vacuum in the MWAB method (atmospheric processing), despite lower temperatures and shorter processing times, may account for the slightly higher p‐AV value than the IB method. In contrast, the PV remained unaffected.

#### 3.3.4 The Effect of MWAB on Heavy Metals

There is no information about the effect of MWAB on heavy metals in vegetable oils. Sarafraz Ardakani et al. (2023) used MW (600 W) at different temperatures and times to digest sesame oil during oil refining. The authors reported that metal cations (Al, K, Fe, Pb, Cd, Ni, Cu, Cr, and Co) were removed via interaction with bleaching clays. Post‐bleaching analysis revealed elevated concentrations of Mg, Ca, Pb, and P ions. This observation suggests potential contributions from the wash water and adsorbents used in the bleaching process. Furthermore, the incomplete removal of these elements during the neutralization stage may have contributed to increased calcium and magnesium levels.

#### 3.3.5 Effect of MWAB on Clay and Energy Consumption

Oil bleaching using MWAB can occur at temperatures below 100°C for a short period (8 min). These advantages are attributed to the low specific heat capacity and high bleaching efficiency. Thus, the MWAB approach can reduce energy consumption by reducing process time and temperature, and eliminating the need for a vacuum compared to the IB. Seçilmiş et al. (2021b) revealed that sunflower oil bleaching by MW (80 W, < 100°C, 8 min) lowered the clay by about 50% and the bleaching time by 73%.

According to the heatmap, B.E demonstrates strong positive correlations with bleaching clay (*r* = 0.88) and time (*r* = 0.72). In contrast, a moderate positive correlation was observed between B.E and power (*r* = 0.48).

### 3.4 Membrane–Assisted Bleaching (MAB)

**FIGURE 5:**
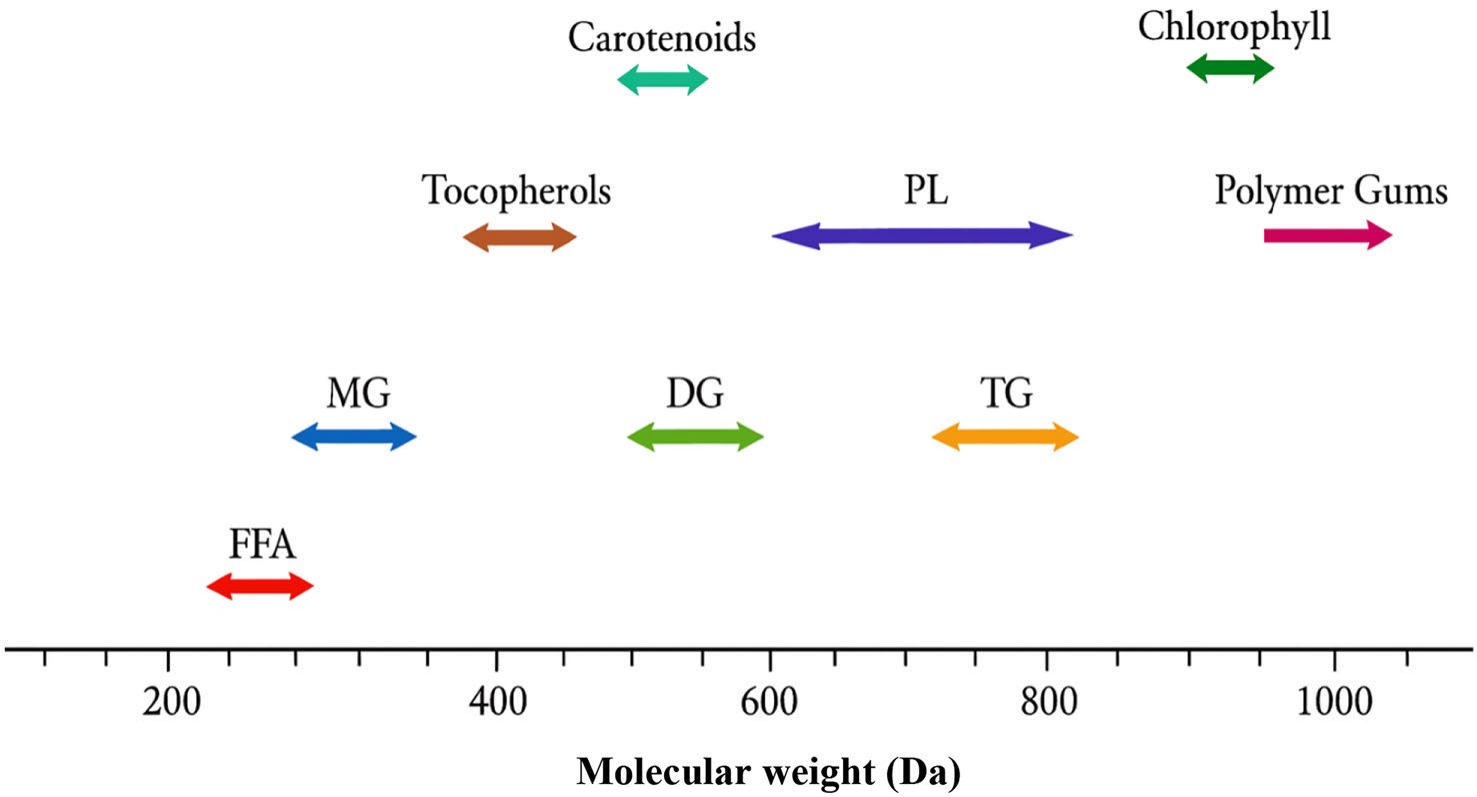
Different sizes of various compounds in oils bleached with MAB.

MB technology, a non‐thermal separation process, efficiently separates various components through a size exclusion mechanism (Table 5). Many researchers are exploring the replacement or improvement of traditional bleaching methods with more cost‐effective alternatives, such as MB technology. Membrane processing offers a simpler and more advantageous alternative to IB, characterized by low energy consumption, ambient operation, the absence of chemical additions, and the preservation of nutrients and desirable oil components (Manjula and Subramanian 2006). Additionally, it helps reduce oil loss and decreases wastewater production (Dadfar et al. 2020). Pressure‐driven membrane technologies include reverse osmosis (RO), nanofiltration (NF), ultrafiltration (UF), and microfiltration (MF), each designed to separate solutes based on their size or particles to be separated (Figures 1D and 5). These processes are implemented using commercially available membrane devices in four standard designs: plate‐and‐frame, tubular, spiral‐wound, and hollow‐fiber (Manjula and Subramanian 2007). Membrane‐based vegetable oil processing has been the subject of considerable investigation, employing both polymeric and ceramic membranes. Studies have explored both solvent‐based and solvent‐free approaches. While solvent‐free methods provided insights into the fundamental mechanisms of membrane rejection and constituent separation, the resulting low permeate fluxes hindered their industrial applicability (Rangaswamy et al. 2021).

**TABLE 5:** The effect of membrane‐assisted bleaching (MAB) on the bleached qualities of different types of oil.

Type of oil	MAB condition	Chlorophyll (mg/kg)			Lovibond color					Ref.
					Crude		Permeate			
		Feed Permeate	Feed Permeate	Rejection* %	R	Y	R	Y	Rejection* %	
Model oil (refined sunflower oil + chlorophyll) Model oil (refined sunflower oil‐hexane solution + chlorophyll) Crude soybean oil	Nonporous membranes—undiluted oils NTGS‐ 2100 NTGS‐1100 Polyethylene microfiltration membrane (PE‐30) NTGS‐ 2100 NTGS‐1100 NTGS‐2100			95.6 72.3 3.1 65.9‐77 23.4‐33.2					9.7‐69.5	(Reddy et al. 2001)
Model oil (refined sunflower oil + lutein) Model oil (refined sunflower oil + chlorophyll) Model oil (refined sunflower oil +*β*‐carotene)	Nonporous membranes—undiluted oils NTGS‐2200, Silicon, Flat sheet, Stirred cell NTGS‐2200, Silicon, Flat sheet, Stirred cell NTGS‐2200, Silicon, Flat sheet, Stirred cell	54.0	0.5	99.2						Subramanian et al. 2001
Sunflower oil	Nonporous membranes—undiluted oils NTGS‐2200, Silicon, Flat sheet, Stirred cell				0.9	7.7	0.2	1.7	77.9	(Subramanian et al. 1998)
Rice bran oil Rice bran oil, 25% miscella (w/w) Soybean oil (degummed), 33% miscella (w/w)	Nonporous membranes—undiluted oils NTGS‐2200, Silicon, Flat sheet, Stirred cell Nonporous membranes—hexane‐diluted oils NTGS‐2200 NTGS‐2200 Silicon, Flat sheet, Stirred cell				1.0 1.0	7.0 7.0	0.5 1.0	0.6 0.7	52.5 74.0	(Saravanan et al. 2006)
Palm oil Palm olein Palm oil, 50% miscella (w/w) Palm olein, 50% miscella (w/w)	Nonporous membranes‐ hexane‐diluted oils NTGS‐2200, Silicon, Flat sheet, Stirred cell NTGS‐2200, Silicon, Flat sheet, Stirred cell NTGS‐2200, Silicon, Flat sheet, Stirred cell NTGS‐2200, Silicon, Flat sheet, Stirred cell				27.4 26.6 25.1 22.1	3.2 2.2 4.1 4.4	24.0 24.1 23.9 20.1	2.1 2.0 4.2 4.3	12.9 9.4 4.6 8.7	(Arora et al. 2006)
Cottonseed oil, 25% miscella Rapeseed oil, 25% miscella	Ultrafiltration membranes—hexane‐diluted oils Type‐E, PS/PA/fluorinated polymer, MWCO: 500Da‐30 kDa, Flat sheet (commercial/laboratory cast), Stirred cell Type‐E, PS/PA/fluorinated polymer, MWCO: 500Da‐30 kDa, Flat sheet (commercial/laboratory cast), Stirred cell	18.3	10.0	45.4	3.6	24.0	0.3	2.4	90.7	(Koseoglu et al. 1990)
Cottonseed oil, 25% miscella	Ultrafiltration membranes—hexane‐diluted oils DS‐7, Thin film polymer, MWCO: 1kDa, Flat sheet, Cross‐flow system				10.0	71.0	2.0	30.0	66.9	(Lin et al. 1997)
Model oil (refined sunflower oil + chlorophyll) Model oil (refined sunflower oil + chlorophyll), 50% miscella (w/w)	Ultrafiltration membranes—hexane‐diluted oils NTGS‐2100, Silicon, Flat sheet, Stirred cell Nonporous membranes—hexane‐diluted oils NTGS‐2100, Silicon, Flat sheet, Stirred cell	NR NR	NR NR	95.6 72						(Kondal Reddy et al. 2001)
Crude rice bran oil	Nonporous membrane NTGS‐2200 membrane NTGS‐2100 membrane				5‐7.4	4.5‐21	1.1‐4.4 1	1.6‐12.3 1.9	26.2‐84.2 84.6	(Manjula and Subramanian 2009)
Crude soybean oil	Ultrafiltration pilot unit using multi‐channel (19 channels) ceramic membrane with an active alumina layer, permeation area of 0.2 m^2^ and mean pore size of around 0.01 μm	2.79	2.28	18.27	3.7		3.6			(Ribeiro et al. 2008)
Olive oil	Ultrafiltration using ETNA10PP membrane			40.42						(Dadfar et al. 2020)

* Chlorophyll rejection.

A large number of studies have predominantly concentrated on the selectivity and productivity of membranes in oil processing applications. However, the long‐term durability of the membrane, ideally exceeding 1–2 years of continuous operation within the hexane‐based process environment, is equally paramount. This necessitates the development of membranes that exhibit high compatibility with hexane and have an extended service life. While some studies have reported stable performance up to 32 days of continuous operation and 414 days of physical aging under periodic monitoring, further advancements are required to achieve the desired longevity. However, membrane endurance has been assessed in only a few studies (Manjula and Subramanian 2006; Rangaswamy et al. 2021).

The experimental methodologies employed in the majority of studies to date have not adequately reflected the operational parameters encountered in industrial‐scale oil processing systems. Specifically, membrane performance has predominantly been evaluated using dead‐end filtration cells, often with full‐strength miscella (25%–30% oil concentration) or, in some instances, with even lower oil concentrations. While this approach may be suitable for providing a preliminary assessment of membrane selectivity, its limited applicability to industrial scenarios warrants further discussion. In contrast, cross‐flow (tangential flow) filtration is a more industrially relevant configuration, and its adoption is strongly recommended. This approach has been demonstrated to effectively mitigate the adverse effects of concentration polarization, leading to improved membrane permeability and separation efficiency. However, careful optimization of the cross‐flow velocity is imperative to ensure optimal performance and prevent any detrimental effects on rejection characteristics (Manjula and Subramanian 2006; Rangaswamy et al. 2021). Industrial membrane operations typically employ partial retentate recirculation to maintain a high flow rate and crossflow velocity, resulting in higher feed concentrations. However, most studies evaluate membrane performance using lower‐concentration refined oil miscella, rather than the higher‐concentration crude miscella found in industrial settings. Only a few studies have tested membranes at industrially relevant concentrations (35%–52%). Therefore, standardized testing should utilize cross‐flow systems with higher‐strength, preferably prefiltered crude oil miscella to better reflect industrial conditions (Manjula and Subramanian 2006; Rangaswamy et al. 2021).

#### 3.4.1 The Effects of MBAB on Pigments and Color

Despite the insufficient size differences between triacylglycerols (TAGs, > 800 Da), carotenoids (< 570 Da), and chlorophyll (892 Da) (Figure 5) to enable size exclusion membrane separation, micelle‐enhanced ultrafiltration successfully rejected these color pigments in various crude oil miscella systems (Table 5) (Rangaswamy et al. 2021). The rejection of color compounds, according to Subramanian et al. (2001), resulted from their entrapment within reverse micelles formed by phospholipids in the miscella. In a study by Koseoglu et al. (1990), five different ultrafiltration (UF) membranes were tested for their ability to simultaneously degum and decolorize crude vegetable oils (cottonseed, peanut, rapeseed, and soybean) (Koseoglu et al. 1990). Two membranes, designated as Type A and Type E, were selected for decolorization. The results display that using micelle‐enhanced ultrafiltration (MEUF) with the Type E membrane showed varying degrees of carotenoid and Lovibond color reduction across the different oil types. Carotenoid rejection was notably high only for cottonseed (82%) and peanut (68%) oils, while chlorophyll reduction showed more consistent results (41%–67%) across all oils tested (Table 5). The results suggested that MEUF is a viable technique for simultaneously degumming and partially decolorizing vegetable oils, potentially reducing the amount of adsorbents required in subsequent bleaching steps. The research conducted by Reddy et al. (2001) focused on the processing of sunflower oil and soybean oil. Batch membrane bioreactor (MBR) systems incorporating three membrane types—NTGS‐2100 and NTGS‐1100 polymeric composite membranes, and a PE‐30 polyethylene microfiltration membrane—were utilized. 96% and 72% of chlorophyll removal efficiencies were observed with the NTGS‐2100 membrane for undiluted oil and a 50% (w/w) oil‐hexane solution, respectively. The results indicate that the polymeric composite MABs achieved greater decolorization (Table 5). In contrast, the MAB of PE‐30 showed minimal rejection of color compounds (Reddy et al. 2001). In a survey by Dadfar et al. (2020), carotenoid and chlorophyll levels were reduced by 71.3% and 40.42%, respectively, using optimized filtration parameters of 3.7 bar, 36.5°C, and 300 rpm (Table 5). However, Subramanian, Nakajima, and Kawakatsu (1998) found that the color reduction observed in the permeate of soybean oil ranged from 74% to 80%. Abdorrezaee and Raisi (2021) concluded that a three‐stage hybrid MB process, integrating ultrafiltration, nanofiltration, and pervaporation, represents a promising approach for removing phospholipids, pigments, and solvents from crude canola oil miscella. This combination of techniques can enhance the efficiency of the purification process, leading to improved oil quality and yield. Higher molecular weight polyethylene glycols (PEGs) significantly enhanced both the selectivity and permeation flux of polyethersulfone nanofiltration membranes during oil bleaching. Using N‐10PEG4000, the MB achieved a 43.56% color reduction in degummed oil at a flux rate of 15.50 L/m^2^·h. Combined with the initial degumming stage, this resulted in an overall color reduction of 91%, demonstrating the potential for a clay‐free bleaching process using a two‐stage MB system (Abdorrezaee and Raisi 2021).

These studies highlight the complex relationship between membrane type, pore size, and efficacy in crude oil processing. A 30% reduction in Lovibond color was observed in crude palm oil using a 9 kDa MWCO PES UF membrane, while a 100 kDa PES UF membrane resulted in a 25% color reduction in soybean oil (Manjula and Subramanian 2006).

#### 3.4.2 Sterols and Tocopherols

The study conducted by Dadfar et al. (2020) indicated a general reduction in sterol levels of olive oil treated with the MAB. When comparing total sterol content, the MAB method exhibited the highest levels, while the IB process resulted in a decrease. Notably, implementing MAB filtration and IB methods led to an increase in β‐sitosterol content. Among the various oils analyzed, campesterol levels were lowest in the MAB‐treated oil and highest in the IB method. The ratio of campesterol to stigmasterol, which serves as an essential quality index for olive oil, was higher in crude and MAB than in the IB method. However, the difference between the crude oil and MAB was not statistically significant. Furthermore, using microfiltration to separate vesicle‐like and soap‐derived macrostructures in lampante olive oil reduced overall sterol content, with elimination rates ranging from 36% to 50%. Stigmasterol experienced only a minimal reduction in the study by Hafidi et al. (2005). The research obtained by Arora et al. (2006) focused on refining processes and nutrient retention, and assessing the performance of a composite polymeric MB composed of nonporous materials for the treatment of both undiluted and hexane‐diluted crude palm oil and palm olein. These oils are rich in natural antioxidants, particularly tocopherols and tocotrienols, with crude palm oil typically containing between 600 and 1000 mg/kg of these compounds. Conventional refining generally preserves only about 50% of these antioxidants. No significant selectivity for tocopherols and tocotrienols was demonstrated by MAB filtration, irrespective of hexane dilution of the feed. Under optimal bleaching conditions (0.7% bleaching clay, 3.7 bar, 36.5°C, 300 rpm), a 57.12% increase in specific bioactive compounds, including phenolic compounds, was observed in the filtered olive oil (Dadfar et al. 2020).

Findings obtained by Subramanian, Nakajima, and Kawakatsu (1998) on crude soybean and rapeseed oils, it was demonstrated that composite MABs made from polymers could selectively remove color pigments, phospholipids, and certain oxidation products without the use of organic solvents, while allowing tocopherols to pass through. This process resulted in a 12%–15% increase in tocopherol concentration within the permeate. Moreover, no tocopherol changes were observed by Ribeiro et al. (2008).

#### 3.4.3 Primary and Secondary Oxidative Compounds

In the study by Dadfar et al. (2020) on olive oil bleaching using 0.7 wt.% bleaching clay, it was found that under optimal filtration conditions—specifically, a pressure of 3.7 bar, a temperature of 36.5°C, and a stirring rate of 300 rpm—the oil processed with the MAB demonstrated significantly more significant reductions in acid value and TBA. The reductions were reported around 12.42% and 14.46%, respectively, notably higher than those achieved through IB. Subramanian, Nakajima, and Kawakatsu (1998) reported that the reduction of oxidation products in crude soybean and rapeseed oils ranged from 50% to 87%. During oil bleaching, triglyceride dimerization occurs. This process produces conjugated dienes and trienes. Conjugated dienes have an absorption band at approximately 232 nm, while trienes absorb at around 270 nm. The presence of these conjugated dienes and trienes renders the oil more prone to further oxidative reactions. Reddy et al. (2001) revealed that membrane processing of conventionally bleached oil mitigated oxidative degradation by preventing the formation of conjugated dienes and trienes under mild operating conditions. The results indicated slight rejection of these compounds, suggesting a lower membrane affinity and permeability for conjugated double bonds in fatty acids compared to their unconjugated counterparts.

The provided heatmap illustrates the correlation coefficients among five variables: pressure, temperature, stirring rate, and pigments. Pressure exhibits a weak positive correlation with chlorophyll (*r* = 0.17) and carotenoids (*r* = 0.02), which are less effective than other treatments.

### 3.5 Adsorption Kinetics and Thermodynamics

In recent years, adsorption kinetics and thermodynamic properties related to the adsorption of constituents of edible oils onto adsorbent surfaces have garnered considerable attention. Despite limited reports on this subject, studies have investigated the kinetics, isotherms, and thermodynamic properties during the bleaching process of several oils, including rice bran oil (Pohndorf, Cadaval Jr, and Pinto 2016), palm oil (Almeida et al. 2019; Silva et al. 2014), olive oil (Asgari et al. 2018), and soybean oil (Abedi et al. 2021; Maleki et al. 2024).

#### 3.5.1 Adsorption Isotherms, Kinetics, and Thermodynamic Studies

Adsorption at the interface between a solid and a liquid is considerably more intricate than at a gas–solid interface because solvent molecules and the potential for strong interactions cover the surface. Consequently, solvent displacement is required for adsorption to occur (Sahoo and Prelot 2020). The adsorption process comprises attaching components (adsorbate) from a gas or a liquid phase to the solid surface (adsorbent) in two ways: (1) physisorption occurs as a result of weak van der Waals interactions, and (2) chemisorption involves forming a strong bond such as ionic or covalent, among the adsorbent and adsorbate (Abedi et al. 2021; Saleh 2022). The amount of adsorbent, the adsorbate concentration, temperature, contact time, pH, stirring speed, specific surface area, particle size, and porosity affect the sorption process. Adsorption reactions occur rapidly initially and gradually slow down as equilibrium is approached. However, the time required to reach equilibrium depends on the concentration of absorbate and adsorbent, as well as the characteristics of the liquid phase (Oladipo et al. 2021). Understanding the mechanism of impurity removal from edible oils during IB requires knowledge of adsorption isotherms, kinetics, and thermodynamics (Abedi et al. 2021; Saleh 2022).

#### 3.5.2 Adsorption Kinetics Models

The rate of adsorption is controlled by adsorption kinetics, which directly affects the duration needed for the process to achieve equilibrium (Sahoo and Prelot 2020). Several kinetic models, including pseudo‐first‐order, pseudo‐second‐order, Elovich, and Weber's intraparticle diffusion (Table 6), are commonly used to analyze the adsorption dynamics (Sahoo and Prelot 2020). During the early adsorption phases, the Lagergren pseudo‐first‐order model suggests that the solute uptake rate is directly related to the difference between the saturation concentration and the quantity of solute absorbed as time progresses. In the bleaching of soybean oil using activated bentonite clay, the pseudo‐first‐order model best described the adsorption kinetics of carotenoids and chlorophyll, indicating a significant physisorption mechanism with some chemisorption contribution, rather than being primarily diffusion‐controlled (Abedi et al. 2021). Similarly, in olive oil bleaching with activated bentonite clay, the pseudo‐first‐order model best describes the carotenoid adsorption kinetics in both the IB (k_1_ = 0.0822 min^−1^, *R* ^2^ = 0.9728) and USAB methods (k_1_ = 0.1714 min^−1^, *R* ^2^ = 0.9777), indicating physisorption. Conversely, chlorophyll removal followed pseudo‐second‐order kinetics in IB (k_2_ = 0.3463 g·mg^−1^·min^−1^, *R* ^2^ = 0.9805) and USAB (k_2_ = 0.2901 g·mg^−1^·min^−1^, *R* ^2^ = 0.9958), confirming chemisorption as the dominant mechanism (Asgari et al. 2018). The pseudo‐second‐order model is based on the premise that the rate‐determining stage is chemisorption, enabling it to predict behavior throughout the entire adsorption process. In general, the first‐order kinetic equation explains the diffusion phase, whereas the second‐order kinetic equation pertains to the adsorption phase at active sites (Saleh 2022). The kinetic study of rice bran oil bleaching demonstrated distinct adsorption mechanisms for pigment removal (carotenoids and chlorophylls). Activated earth followed pseudo‐second‐order kinetics, indicating chemisorption through electrostatic interactions, whereas chitin and chitosan exhibited pseudo‐first‐order kinetics, suggesting physical adsorption dominated by concentration gradients (Pohndorf, Cadaval Jr., and Pinto 2016). The Elovich kinetic model, which assumes an energetically heterogeneous surface, is commonly employed to analyze adsorption kinetics. It effectively describes second‐order kinetics and the chemisorption mechanism in adsorption processes (Sahoo and Prelot 2020; Saleh 2022). The Elovich kinetic model still showed good correlation (*R* ^2^ = 0.82–0.96) for the adsorption of trace metals (Fe(II)/Cu(II)) and pigments on bentonite clay under HVEF bleaching. The increasing initial adsorption rates at higher voltages (20 kV) suggested energetically heterogeneous adsorption sites, consistent with the Freundlich isotherm's multilayer adsorption behavior (Abedi, Roohi, et al. 2020). The model of Weber and Morris explains the diffusion process involving mass transfer within particles. The Weber and Morris model describes mass transfer occurring within particles (intraparticle diffusion) or, in other words, identifies the diffusion mechanism in the adsorption. This model is an internal diffusion model for determining the rate‐controlling step, assuming that the adsorbate's diffusion through the adsorbent represents the slowest stage in the process (Saleh 2022; Steffens et al. 2024). In the case of palm oil bleached with silica‐smectite composites, the best‐fitting model combined second‐order kinetics and intraparticle diffusion, indicating a complex adsorption mechanism involving both chemisorption and diffusion (Kepdieu, Tchanang, Njimou, Djangang, Maicaneanu, and Tizaoui 2024). Internal diffusion models differ from external diffusion models in that they identify the adsorbate's diffusion through the adsorbent as the rate‐limiting stage. In contrast, adsorption onto active sites and the diffusion of the adsorbate through the liquid layer surrounding the adsorbent occur rapidly and are not considered limiting factors (Saleh 2022).

**TABLE 6:** Kinetic models and their parameters for edible oil/model system bleaching with conventional and novel methods.

Sorption behavior	Model and equation and parameters	Bleaching method/	Sample/Absorbent	Target compounds	Tested models/Best fitting model	Ref.
Kinetic model**s**	(1) Pseudo−first−order $q_{t}=q_{e}\left(1-e^{-k_{1}t}\right)$ (2) Pseudo−second−order $q_{t}=\frac{q_{e}^{2}k_{2}t}{1+q_{e}^{2}k_{2}t}$ (3) Weber and Morris intraparticle diffusion $q_{t}=k_{p}t^{0.5}+C$ (4) Elovich model $q_{t}=\frac{1}{a}\ln\left(1+\mathrm{abt}\right)$ where the parameters in the above equation are: q_e_ and q_t_ (mg g^−1^): adsorption capacities of adsorbent under bleaching methods at equilibrium time and time *t*, respectively. k_1_, k_2_ and k_p_: pseudo‐first‐order rate constant (min^−1^), pseudo‐secondorder rate constant (g mg^−1^ min^−1^), and intraparticle diffusion model rate constant (mg g^−1^ min^−0.5^), respectively. *t* (min): bleaching time C (mg g^−1^): intercept of intraparticle diffusion model. *a*: parameter of the Elovich model associated with the initial velocity (mg kg^−1^ min^−1^) *b*: desorption constant (mg kg^−1^).	IB and USAB methods	Soybean oil/activated bentonite clay	Carotenoid and chlorophyll and heavey metals	Models (1–3)/Pseudo‐first‐order model	(Elahe Abedi et al. 2021)
		IB and HVEFAB methods	Soybean oil/activated bentonite clay	Carotenoid and chlorophyll	Models (1–4)/Pseudo‐first‐order model	(Abedi, Amiri, et al. 2020)
		IB and USAB methods	Olive oil/activated earth	Carotenoid and chlorophyll	Models (1–3)/Pseudo‐first‐order (carotenoids) & Pseudo‐second order (chlorophylls)	(Asgari et al. 2018)
		IB method	Palm oil/acid‐activated and neutral earths	Carotenes	Models (1–3)/Pseudo−first−order & Intra‐particle diffusion	(Almeida et al. 2019)
		IB method	Rice bran oil/activated earth, chitosan and chitin	Chlorophylls and carotenoids, and peroxides	Models (1&2)/Pseudo‐second‐order (activated earth); Pseudo‐first‐order model (chitin and chitosan)	(Pohndorf, Cadaval Jr., and Pinto 2016a)
		IB method	Palm oil/composites based silica‐smectite	β‐carotene	Models (1–3)/Second‐order and intraparticle diffusion	(Kepdieu, Tchanang, Njimou, Djangang, Maicaneanu, and Tizaoui 2024)
		IB method	Oil from catfish waste/95% activated earth and 5% activated carbon (w/w)	Carotenoids and peroxides	Models (1, 2 &4)/Pseudo‐first order and pseudo‐second order models	(Igansi et al. 2019)
		IB method	Olive pomace oil/activated carbon–clay composite	Acid blue 29 and Methylene blue	Models (1&2)/Pseudo‐second‐order	(Marrakchi et al. 2017)
		IB method	Rice bran oil bleaching/Activated earth	Carotenoids and chlorophylls	Models (1, 2 &4)/Pseudo‐second order model	(Pohndorf, Pinheiro, and Pinto 2016)
		IB method	Shea butter and palm oil/Acid‐activated Cameroonian smectite	Pigments and free fatty acids	Models (1, 2 &3)/Pseudo‐second order	(Baptiste et al. 2020)
		Adsorption and desorption strategy	Crude palm oil/five adsorbents	Recovering β‐carotene in the refining process	Models (1–3)/Pseudo−second−order	(Steffens et al. 2024)
		IB method	Cotton seed oil/Organic acid‐activated carbon	Pigments	Models (2&3)/Both pseudo‐second order and intra‐particle diffusion	(Chetima et al. 2018)
		IB method	Chloroform/different macroporous Amberlite resins	β‐carotene	Models (1–3)/Pseudo‐first‐order and intraparticle diffusion kinetic models	(Kurtulbaş et al. 2023)
		IB method	Palm oil/acid activated bleaching earth	Carotenes and phosphorus	Models (1–3)/Both the pseudo‐first‐order and the pseudo‐second‐order	(Silva et al. 2013)
Isotherm studies: Equilibrium models	(1) Langmuir model $q_{e}=\frac{K_{L}q_{m}C_{e}}{\left(1+K_{L}C_{e}\right)}$ (2) Freundlich model qe=KFCe1n (3) Temkin model $q_{e}=\frac{\mathrm{RT}}{b_{T}}\ln\left(A_{T}C_{e}\right)$ (4) Toth model qe=KTCeaT+Ce1t (5) BET model $q_{e}=q_{m}\frac{K_{s}C_{e}}{\left(1-K_{1}C_{e}\right)\left(1-K_{1}C_{e}+K_{s}C_{e}\right)}$ (6) Hill‐de‐Boer isotherm $C_{e}=\left(\frac{q_{e}/q_{m}}{K_{1H}\left(1-\left(q_{e}/q_{m}\right)\right)}\right)\exp\left(\left(\frac{q_{e}/q_{m}}{\left(1-\left(q_{e}/q_{m}\right)\right)}\right)-\left(\frac{K_{2H}\left(q_{e}/q_{m}\right)}{\mathrm{RT}}\right)\right)$ (7) Multi‐component Freundlich type isotherm $q_{i}=K_{i}C_{i}{\left(\sum_{j=1}^{k}a_{\mathrm{ij}}C_{j}\right)}^{n_{i}-1}$ where the parameters in the above equation are: q_e_ and q_m_ (mg g^−1^): amount of adsorbate adsorbed in solid phases (adsorbent) and maximum adsorption capacity, respectively. C_e_: amount of bleaching clay in the solution (mg L^−1^). K_F_: Freundlich coefficient corresponded to the adsorption capacity ((mg g^−1^) (L mg^−1^)^1/n^), n: constant corresponded to the adsorption intensity. K_L_: Langmuir coefficient corresponded to the energy of adsorption (L mg^−1^). b_T_: Temkin constant corresponded to the heat of sorption (J mol^−1^) A_T_: Temkin isotherm constant related to the maximum binding energy (L mg^−1^), T and R: absolute temperature (K) and the universal constant (8.314 J mol^−1^ K^−1^), respectively. a_T_: Toth isotherm constant (L mg^−1^), K_T_: maximum adsorption capacity for Toth model (mg g^−1^), t: a parameter, in the term (1/t), shows the heterogeneity of the adsorbent In BET model, K_s_ (g/mg) and K_l_ (g/mg): equilibrium adsorption constants for the first layer and upper layers, respectively. In Hill‐de‐Boer, K_1H_ and K_2H_ (J.mol^−1^): adsorbent‐adsorbate and adsorbate‐adsorbate interaction, respectively. In Multi component isotherms, a_ij_ (kg/mg): inhibition to the adsorption of component i by component j.	IB and USAB methods	Soybean oil/activated bentonite clay	Carotenoid and chlorophyll and heavey metals	Models (1–4)/Toth isotherm model	(Elahe Abedi et al. 2021)
		IB and HVEFAB methods	Soybean oil/activated bentonite clay	Carotenoid and chlorophyll	Models (1&2)/Freundlich model	(Abedi, Amiri, et al. 2020)
		IB and USAB methods	Olive oil/activated earth	Carotenoid and chlorophyll	Models (1&2)/Langmuir (carotenoids) & Freundlich (chlorophylls)	(Asgari et al. 2018)
		IB and USAB methods	Canola oil/Acid‐activated bleaching earth	Carotenes	Models (2)/Freundlich	(Icyer and Durak 2018)
		IB and USAB methods	Rapeseed oil/acid‐activated bentonite	Carotenes	Models (2)/Freundlich	(Su et al. 2013)
		IB method	Palm oil/acid‐activated and neutral earths	Carotenes	Models (1, 2 &5)/Langmuir model	(Almeida et al. 2019)
		IB method	Palm oil/composites based silica‐smectite		Models (1&2)/Freundlich isotherm	(Kepdieu, Tchanang, Njimou, Djangang, Maicaneanu, and Tizaoui 2024)
		IB method	Oil from catfish waste/95% activated earth and 5% activated carbon (w/w)	Carotenoids and peroxides	Models (1&2)/Langmuir model	(Igansi et al. 2019)
		IB method	Olive pomace oil/activated carbon–clay composite	Acid blue 29 and Methylene blue	Models (1&2)/Langmuir model	(Marrakchi et al. 2017)
		IB method	Rice bran oil bleaching/Activated earth	Carotenoids and chlorophylls	Models (1&2)/Freundlich model	(Pohndorf, Cadaval Jr., and Pinto 2016)
		IB method	Shea butter and palm oil/Acid‐activated Cameroonian smectite	Pigments and free fatty acids	Models (1–3)/Freundlich (pigments) and Temkin (free fatty acids)	(Baptiste et al. 2020)
		Adsorption and desorption strategy	Crude palm oil/five adsorbents	Recovering β‐carotene in the refining process	Models (1,2&6)/Both Hill de Boer and Langmuir fitted well the equilibrium experimental data	(Steffens et al. 2024)
		IB method	Cotton seed oil/Organic acid‐ activated carbon	Pigments	Models (1&2)/Langmuir isotherm	(Chetima et al. 2018)
		IB method	Chloroform/different macroporous Amberlite resins	β‐carotene	Models (1–3)/Freundlich and Temkin models	(Kurtulbaş et al. 2023)
		IB method	Palm oil/acid activated bleaching earth	Carotenes and phosphorus	Models (1–3, 7)/Langmuir and Freundlich models for β‐carotene and phosphorus	(Silva et al. 2013)
Thermodynamic parameters	(1) van't Hoff equilibrium equation $\ln k_{d}=\frac{∆S^{\circ }}{R}-\frac{∆H^{\circ }}{\mathrm{RT}}$ (2) Standard Gibbs Free Energy $∆G^{\circ }=-\mathrm{RT}\ln k_{d}$ (3) Standard Gibbs Free Energy $∆G^{\circ }=∆H^{\circ }-T∆S^{\circ }$ (4) Clausius‐Clapeyron isosteric heat of adsorption $\ln C_{e}=\frac{∆H_{a}}{\mathrm{RT}}+c$ where the parameters in the above equation are: k_d_: equilibrium constant (dimensionless), R: ideal gas constant (8.314 J mol^−1^ K^−1^) T: temperature (K) ΔH^o^: standard enthalpy, ΔS^o^: standard entropy, and ΔG^o^: standard free energy ΔHa (mol^−1^ kJ): heat in the adsorption, at a constant surface area, or isosteric heat C_e_: adsorbate concentrations (mg kg^−1^)	IB and USAB methods	Soybean oil/activated bentonite clay	Carotenoid and chlorophyll and heavey metals	Eqs 1–3/ Endothermic/Spontaneous between 35°C and 65°C	(Abedi et al. 2021)
		IB and HVEFAB methods	Soybean oil/activated bentonite clay	Carotenoid and chlorophyll	Eqs 1–3/ Endothermic and spontaneous between 35°C and 65°C	(Abedi, Amiri, and Sahari 2020)
		IB and USAB methods	Olive oil/activated earth	Carotenoid and chlorophyll	Eqs 1&2/ Endothermic and spontaneous	(Asgari et al. 2018)
		IB and USAB methods	Rapeseed oil/acid‐activated bentonite	Carotenes	Eqs 1/ Endothermic	(Su et al. 2013)
		IB method	Palm oil/acid‐activated and neutral earths	Carotenes	Eqs 1&2/ Endothermic process	(Almeida et al. 2019)
		IB method	Palm oil/composites based silica‐smectite	β‐carotene	Eqs 1&2/ Endothermic process Physisorption	(Kepdieu, Tchanang, Njimou, Djangang, Maicaneanu, and Tizaoui 2024)
		IB method	Oil from catfish waste/95% activated earth and 5% activated carbon (w/w)	Carotenoids and peroxides	Eqs 1, 2 &4/ Endothermic	(Igansi et al. 2019)
		IB method	Rice bran oil bleaching/Activated earth	Carotenoids and chlorophylls	Eqs 1, 2 &4/ Endothermic and spontaneous processes, and the isosteric heat of adsorption, indicated that the activated surface of the earth was heterogeneous.	(Pohndorf, Cadaval Jr., and Pinto 2016)
		IB method	Shea butter and palm oil/Acid‐activated Cameroonian smectite	Pigments and free fatty acids	Physisorption	(Baptiste et al. 2020)
		Adsorption and desorption strategy	Crude palm oil/five adsorbents	Recovering β‐carotene in the refining process	Eqs 1&2/ Exothermic and spontaneous, physical bonding	(Steffens et al. 2024)
		IB method	Chloroform/different macroporous Amberlite resins	β‐carotene	Eqs 1&2/ Endothermic, non‐spontaneous, and physical adsorption.	(Kurtulbaş et al. 2023)
		IB method	Palm oil/acid‐activated bleaching earth	Carotenes and phosphorus	Eqs 1, 2 &4/ Endothermic, spontaneous, and heterogeneous	(Silva et al. 2013)

#### 3.5.3 Isotherm Adsorption Models

An adsorption isotherm describes the equilibrium relationship at a constant temperature between the amount of adsorbate in the surrounding phase and the amount adsorbed onto the adsorbent's surface. It is essential for understanding solute‐adsorbent interactions and optimizing the use of adsorbents (Saleh 2022). Various isotherm models, such as the Freundlich, Langmuir, Temkin, Hill de Boer, Toth, and BET, are employed to study the adsorption mechanisms involved in the bleaching process of edible oils (Ayawei et al. 2017; Saleh 2022). Table 2 presents the kinetic models and their corresponding parameters for edible oil/model system bleaching using conventional and novel methods.

The Freundlich and Langmuir isotherm equations are frequently used because of their ease of application (Oladipo et al. 2021). Langmuir adsorption is relevant for forming monolayer adsorption on a uniform surface and within the pores of the adsorbent, provided there are no interactions among the adsorbed species. As a result, the adsorbate spreads uniformly across the adsorbent's surface, maintaining consistent enthalpy and adsorption activation energy. No additional adsorption will occur when all active surface sites are filled with adsorbate molecules or ions (Oladipo et al. 2021; Ray et al. 2020). The Langmuir isotherm model best describes the adsorption of acid blue 29 (AB 29) and methylene blue (MB) onto the activated carbon–clay composite, confirming monolayer coverage on homogeneous surfaces. This was evidenced by high *R* ^2^ values (> 0.97) and maximum capacities of 104.83 mg/g (AB 29) and 178.64 mg/g (MB) at 30°C (Marrakchi et al. 2017). The Toth isotherm broadens the scope of the Langmuir model to include heterogeneous processes that display non‐ideal behavior, such as multilayer adsorption or interactions between adsorbate molecules. Consequently, Toth created an equation assuming that the adsorption energies at most sites are less than the average energy (Saleh 2022). In soybean oil bleaching using activated bentonite clay, the Toth isotherm model best describes the adsorption equilibrium of carotenoids and chlorophyll, confirming heterogeneous surface adsorption behavior (Abedi et al. 2021). The Freundlich isotherm explains reversible, non‐ideal, and multilayer adsorption in non‐uniform systems, where the sorbent agent contains varied active sites with varying binding energies. Unlike monolayer models, this isotherm accounts for the formation of multiple adsorbate layers on the surface of the adsorbent (Ray et al. 2020; Sahoo and Prelot 2020). This was demonstrated in the ultrasonic bleaching of rapeseed oil using acid‐activated bentonite, where the Freundlich model provided a better fit (*R* ^2^ = 0.8441 at 25°C and *R* ^2^ = 0.9447 at 130°C) compared to the Langmuir model, indicating its suitability for describing the adsorption of carotenes under varied conditions (Su et al. 2013). The Temkin isotherm model proposes that the heat of adsorption for adsorbate molecules declines linearly as the sorbent surface coverage increases. It describes adsorption as a process involving an even spread of binding energies, capped by the highest energy level. Additionally, the model assumes multilayer adsorption and accounts for indirect interactions among the adsorptive material and the adsorbed molecules (Ray et al. 2020; Saleh 2022). For example, in the adsorption of β‐carotene onto macroporous resins, the Temkin model was applied, and the calculated Temkin isotherm constant values (< 8 kJ mol^−1^) confirmed a physisorption mechanism driven by Van der Waals interactions (Kurtulbaş et al. 2023). The Hill‐de Boer isotherm describes the binding energy of different adsorbing compounds on a uniform adsorbent surface. It relies on the concept that the adsorption mechanism includes cooperative interactions among the adsorbate molecules on the sorbent surface (Ayawei et al. 2017; Ray et al. 2020). For example, in β‐carotene recovery, this model's fit highlights cooperative adsorbate interactions (Ayawei et al. 2017). The BET adsorption isotherm, a theoretical model for multilayer physical sorption, was introduced by Brunauer, Emmett, and Teller. The assumptions of the BET isotherm theory include homogeneous multilayer adsorption due to physisorption, uniform and independent adsorption sites on the solid surface, varying adsorption energies for different layers, equal rates of adsorption and desorption for each layer, and the potential for the formation of multilayers after the monolayer stage (Saleh 2022). The BET model was applied to analyze the multilayer adsorption of carotenes from palm oil using acid‐activated and neutral bleaching earths. Still, the Langmuir model provided the best fit for the adsorption data (Almeida et al. 2019).

#### 3.5.4 Adsorption Thermodynamics

The potential of the adsorption process is influenced by two key factors: the rate of adsorption and the quantity adsorbed. However, to determine if a process is spontaneous or non‐spontaneous, it is essential to take into account the thermodynamic parameters (Table 6) (Ray et al. 2020). Key thermodynamic variables (Table 6), such as the changes in Gibbs free energy (ΔG°), standard enthalpy (ΔH°), standard entropy (ΔS°), isosteric heat of adsorption (ΔH_a_), and activation energy (E_a_), provide insights into the mechanisms of adsorption (Ayawei et al. 2017; Saleh 2022). To comprehend the impact of heat on adsorption mechanisms and determine whether the processes occur spontaneously, thermodynamic parameters are required (Saleh 2022). The thermodynamic analysis revealed that the adsorption of carotenoids and chlorophyll onto activated bentonite clay was endothermic (ΔH° = 50.21–119.75 kJ/mol) and spontaneous (ΔG° = −27.02 to −43.04 kJ/mol at 35°C–65°C), with increasing randomness at the solid–liquid interface (ΔS° = 267.29–476.72 J/mol·K), confirming the feasibility of the bleaching process (Abedi et al. 2021). Higher temperatures enhance the kinetic energy of solutes, accelerating the diffusion of adsorbates. This, in turn, significantly affects the adsorption equilibrium and alters the associated thermodynamic properties (Ray et al. 2020). As demonstrated by Kepdieu, Tchanang, Njimou, Djangang, Maicaneanu, and Tizaoui (2024) in crude palm oil, increasing the temperature from 60°C to 95°C reduced the Gibbs free energy, confirming the enhanced spontaneity of β‐carotene adsorption. Meanwhile, positive enthalpy and entropy values indicated an endothermic process driven by increased molecular randomness at the adsorbent–oil interface.

ΔG° is utilized to determine the spontaneity and favorability of the adsorption procedure. A negative ΔG° (ΔG < 0) indicates spontaneity and feasibility, while a positive ΔG° (ΔG > 0) suggests that the process is unfeasible and not spontaneous (Saleh 2022). In a spontaneous process, the plot of ΔG° against temperature (T) is consistently linear, and the slope and intercept of the graph allow for the determination of ΔS° and ΔH° values (Ray et al. 2020). ΔH° of adsorption is the heat released or absorbed during the adsorption procedure. The Van't Hoff equation is typically used to calculate ΔH°. A ΔH° value that is negative represents an exothermic adsorption procedure, while a positive ΔH° value suggests an endothermic procedure (Saleh 2022). Lower ΔH° values (approximately 5–40 kJ·mol−1) often indicate physisorption, whereas elevated levels (40–800 kJ.mol^−1^) correspond to chemisorption due to the strength of chemical bonds (Ayawei et al. 2017). In the study on soybean oil bleaching, the adsorption of carotenoids (ΔH° = 50.21–119.75 kJ·mol^−1^) and chlorophyll (ΔH° = 69.3–104.84 kJ·mol^−1^) onto activated bentonite clay exhibited chemisorption characteristics, as evidenced by the high enthalpy values (Abedi et al. 2021). The Van't Hoff equation is used to determine ΔS° during adsorption. A positive ΔS° indicates the adsorbent's attraction for the adsorbate and suggests enhanced disorder at the solid/liquid interface, along with alterations in the adsorbent's and adsorbate's structure (Saleh 2022). A positive ΔS° value arises from the energy reallocation between the adsorbent and the substance being adsorbed (Steffens et al. 2024). The adsorption of carotenes and phosphorus onto acid‐activated bleaching earth was found to be a spontaneous (ΔG° < 0) and endothermic (ΔH° > 0) process with positive entropy values (ΔS° = 0.40 and 0.19 kJ·mol^−1^·K^−1^, respectively), indicating an entropy‐driven adsorption mechanism where increased temperature enhances adsorption capacity (Silva et al. 2013). Isosteric heat of adsorption (∆H_a_), or differential enthalpy of adsorption, is defined as the proportion of minor alterations in the adsorbate's enthalpy to slight variations in the quantity adsorbed, all measured under constant temperature conditions. Simply, it reflects the heat involved in adsorption when the amount of adsorbate is kept constant. In homogeneous adsorbents, ∆H_a_ of adsorption is constant regardless of the adsorbate amount, while in heterogeneous adsorbents, ∆H_a_ varies with adsorbate quantity. The Clausius‐Clapeyron equation calculates ∆H_a_ for characterizing adsorption processes (Saleh 2022). In the soybean oil bleaching study, thermodynamic analysis revealed decreasing ΔH_a_ with surface coverage for chlorophyll adsorption (ΔH° = 69.3–104.84 kJ·mol^−1^) and carotenoids (ΔH° = 50.21–119.75 kJ·mol^−1^) onto activated bentonite clay, confirming surface heterogeneity (Abedi et al. 2021). E_a_ represents the minimum energy required to initiate a chemical reaction, and it can be predicted for adsorption processes using the Arrhenius Equation. A negative E_a_ value indicates low temperature and an exothermic sorption process, while a positive E_a_ value means that energy (increased temperature) is needed, indicating an endothermic process (Saleh 2022). Igansi et al. (2019) report positive Ea values of 47.1 kJ mol^−1^ for carotenoids and 44.82 kJ mol^−1^for peroxides, confirming that adsorption in catfish oil bleaching is endothermic, with higher temperatures (60°C–80°C) enhancing adsorption capacity by activating more adsorbent sites. The endothermic nature of the process suggests that energy is needed to overcome the energy barrier for adsorption, which is consistent with the physical interactions between the adsorbent and adsorbate, as further supported by the thermodynamic parameters (ΔH < 40 kJ mol^−1^).

## 4 Conclusion and Future Trends

This study comprehensively reviewed the effects of emerging bleaching techniques, including US‐, HVEF‐, MW‐, and MB‐assisted bleaching methods, in comparison to IB on bioactive compounds (sterols and tocopherols), pigments, oxidative indices, heavy metals, and clay consumption of edible oils. This review showed that the novel methods demonstrate various mechanisms to reduce temperature and clay consumption. Color reduction methods rely on either pigment absorption or pigment separation. They can be performed at ambient temperature or below typical IB temperatures to minimize adverse effects associated with high temperature and prolonged processing. Additionally, different patterns were observed to either degrade or protect the bioactive compounds and prevent oil oxidation. Each technique offers advantages and drawbacks, and importantly, they can be produced at industrial scales. To reduce chlorophyll and carotenoid pigments, the use of bleaching clay is necessary for oil bleaching. Nevertheless, except for MBAB, combining these novel methods with bleaching clay yields a reduction in the quantity of bleaching clay used and mitigates its associated disadvantages. Moreover, the kinetics of edible oil bleaching typically follow pseudo‐first‐order or pseudo‐second‐order models, with intraparticle diffusion limiting the rate. At the same time, thermodynamic analyses indicate that these processes are endothermic and spontaneous, driven by an increase in entropy.

Emerging bleaching technologies face barriers to industrial adoption, including high equipment costs, expertise requirements, and scalability challenges that hinder consistent performance across diverse oils. Future research should focus on developing cost‐effective, modular systems, conducting pilot‐scale validation, and integrating processes that combine ultrasonication with enzymatic or solvent‐based methods to enhance bleaching efficiency and improve oil quality. Life cycle assessments and sustainable practices, such as clay recycling, are also crucial. This review highlights the potential of these technologies to enhance the sustainability of edible oil processing and urges academia and industry to address these gaps through targeted research and pilot projects.

## Author Contributions

**Elahe Abedi:** investigation (equal), methodology (equal), project administration (equal), writing – original draft (equal), writing – review and editing (equal). **Hamid‐Reza Akhavan:** writing – original draft (equal), writing – review and editing (equal). **Seyed Mohammad Bagher Hashemi:** writing – original draft (equal), writing – review and editing (equal). **Najmeh Oliyaei:** writing – original draft (equal), writing – review and editing (equal). **Mahmoud Sourghali:** writing – original draft (equal), writing – review and editing (equal). **Ali Karimzadeh:** writing – original draft (equal), writing – review and editing (equal). **Marzieh Rownaghi:** writing – original draft (equal), writing – review and editing (equal).

## Ethics Statement

This article does not contain any studies with human participants or animals performed by any of the authors.

## Conflicts of Interest

The authors declare no conflicts of interest.

## Data Availability

Research data is not shared.
